# Full-field X-ray fluorescence imaging using a Fresnel zone plate as a coded aperture: optimized reconstruction algorithm and first trial of hyperspectral XANES mapping

**DOI:** 10.1107/S1600577526004789

**Published:** 2026-06-01

**Authors:** Gautier Landrot, Arkadiusz Dawiec, Björn Eckert, Petra Majewski, Michael Brunner, Andrew Fram, Martin Huth, Guillaume Alizon, Claude Menneglier, Enrico Piasentier, Karine Chaouchi, Stéphanie Blanchandin, Pascal Mercere, Martin Chauvin, Emiliano Fonda

**Affiliations:** a Synchrotron SOLEIL L’Orme des Merisiers Départementale 128 91190Saint-Aubin France; b PNDetector, Otto-Hahn-Ring 6, 81739Munich, Germany; c Department of Chemical and Pharmaceutical Sciences University of Trieste Piazzale Europa 1 Trieste TS34127 Italy; Advanced Photon Source, USA

**Keywords:** full-field X-ray fluorescence imaging, hyperspectral XANES mapping, spatial determination of oxydation states, Fresnel zone plate as coded aperture, optimized reconstruction algorithm

## Abstract

This study represents the first feasibility demonstration of hyperspectral X-ray absorption near-edge structure (XANES) mapping performed using a full-field fluorescence imaging approach. This method may be useful in many research fields for determining the spatial distributions of the different oxidation states of an element present at low concentration in X-ray beam-sensitive samples. It was demonstrated that this approach could be easily performed using a full-field imaging method where a Fresnel zone plate (FZP) was employed as a coded aperture, which represented a practical, fast and dose-efficient alternative to the raster-scanning technique, when these two approaches were tested at a beamline not dedicated to X-ray imaging. The basic form of the reconstruction algorithm, which was derived from inline holography, was optimized. This enabled the spatial resolution and overall quality of the reconstructed image to be improved. The ∼62 µm spatial resolution experimentally achieved may be further optimized to about 5 µm using a smaller FZP and larger detector than those used in this investigation. The XANES spectra corresponding to the main chemical species present in the sample, obtained from the tested hyperspectral spectroscopy approach, did not exactly match those expected, as they depended on several empirical processing steps. While these results can be further improved by optimizing the linearity of the detector output via pile-up corrections, further work is needed to establish robustness against variations in masking, background treatment, and intensity-correction choices, especially for studying diluted specimens whose corresponding pixel intensities are similar to those of the background.

## Abstract 2

The full-field X-ray fluorescence imaging method, where a Fresnel zone plate is employed as a coded aperture, was tested for the first time at a beamline, and its corresponding reconstruction code was optimized. It was also employed to perform, also for the first time using full-field and fluorescence modes, hyperspectral XANES mapping.

## 1. Introduction

The oxidation states of an element can be determined by X-ray absorption spectroscopy (XAS) and, more specifically, X-ray absorption near-edge structure (XANES) spectroscopy, which probes the region typically within 30 eV of the absorption edge (Rehr & Albers, 2000 ▸). Determining in 2D the spatial distribution of different oxidation states of an X-ray absorber can be useful in various fields of research, including biochemistry (Carmona *et al.*, 2019 ▸), toxicology (Ortega *et al.*, 2005 ▸), geological and environmental sciences (Kravchenko *et al.*, 2022 ▸; Mosbah *et al.*, 1999 ▸), cultural heritage (Gianoncelli *et al.*, 2024 ▸), electrochemistry (Zhang *et al.*, 2023 ▸), and material (Bohic *et al.*, 2005 ▸) or nuclear sciences (Lechelle *et al.*, 2004 ▸). This can be achieved using hyperspectral XANES mapping, where a series of absorption images are acquired in 2D across the XANES energy range, so that each pixel location in the resulting stack corresponds to a specific XANES spectrum (Pattammattel *et al.*, 2022 ▸; Briois *et al.*, 2024 ▸).

The full-field imaging method employs a large, defocused X-ray beam that entirely illuminates the sample area to image. Until now, full-field X-ray spectroscopic mapping has been limited mostly to a transmission geometry (Briois *et al.*, 2024 ▸), which works best at specific sample thickness and density values, as well as absorber concentration, which should be at least 1 wt% (Kelly *et al.*, 2008 ▸). However, the most relevant applications of hyperspectral XANES mapping concern heterogeneous, complex and often diluted specimens that are encountered in many research fields including environmental, heritage or life sciences. Given the lack of achromatic and effective X-ray optics capable of collecting the emitted X-ray fluorescence (XRF) from an illuminated sample, hyperspectral XANES mapping has been mainly performed in raster scanning, where a micro- or nano-focused X-ray beam is employed to collect, one image pixel at a time and at each energy, an XRF map whose resolution is related to the X-ray probe size (Pattammattel *et al.*, 2022 ▸). This approach may not be adequate when employing bright X-ray beams to probe beam-sensitive and unique materials. Indeed, it has been experimentally observed that a shorter acquisition using a brighter beam may induce more damage to a sample than a longer acquisition with a proportionally less bright beam, for the same total dose of X-rays deposited (Chen *et al.*, 2016 ▸; Jousseaume *et al.*, 2023 ▸). Therefore, from a sample preservation standpoint, the use of a full-field imaging approach is particularly relevant at fourth-generation synchrotrons that typically deliver brighter X-ray beams, including the future upgraded Synchrotron SOLEIL (Nadji & Nadolski, 2023 ▸), compared with synchrotrons of older generations. Although the full-field XRF-XANES mapping approach has not yet been achieved to our knowledge, the imaging method combining full-field and fluorescence modes has emerged over the last years and already been tested at a few beamlines worldwide (Kulow, Guilherme Buzanich *et al.*, 2020 ▸; Siddons *et al.*, 2020 ▸; Zhao *et al.*, 2019 ▸; Klysubun *et al.*, 2023 ▸). A comparison in setup characteristics, energy and spatial resolutions between the imaging methods employed at these beamlines is provided in Table S1 of the supporting information.

When a full-field XRF imaging method is employed, the spatial distributions of multiple chemical elements present in a sample can be individually determined. This requires a bi­dimensional X-ray detector with energy-resolving capability such as the pnCCD-based Color X-Ray Camera (CXC, PNDetector GmbH) (Soltau *et al.*, 2023 ▸). In addition to an X-ray beam illuminating the entire region to image and a bidimensional fluorescence detector, the full-field XRF technique requires the use of an optical device located between the sample and the detector. This is essentially a light mask whose holed part enables the fluorescence emitted from a given sample point source within the field of view to be collected at specific locations on the detector chip. It eventually allows a 2D image to be captured corresponding to the chemical distribution of the fluorescence-emitting elements that are present within the illuminated sample area (Soltau *et al.*, 2023 ▸). Two different types of optical devices have been employed so far to perform full-field X-ray fluorescence imaging at synchrotron facilities, *i.e.* a coded aperture, which had a modified uniformly redundant array (MURA) pattern (Kulow, Guilherme Buzanich *et al.*, 2020 ▸; Siddons *et al.*, 2020 ▸) or polycapillary optic apparatus (Zhao *et al.*, 2019 ▸; Klysubun *et al.*, 2023 ▸) (Table S1). We will not discuss here the possibility of employing a simple pinhole aperture, since this method implies a significant loss of signal on the detector. Coded-apertures, such those featuring a MURA pattern, are known to be inherently more efficient than polycapillary optics when employed as optical apparatus in a full-field fluorescence imaging setup (Kulow, Buzanich *et al.*, 2020 ▸). More recently, a Fresnel zone plate (FZP) has been employed as a coded aperture to perform full-field X-ray imaging in fluorescence mode, using a laboratory setup with a rotating copper anode source (Soltau *et al.*, 2023 ▸). It was demonstrated that this method, combined with a reconstruction approach that involved the use of a contrast transfer function (CTF), was rapid and robust, requiring only a few experimental parameters to perform the image reconstruction step (Soltau *et al.*, 2023 ▸). This contrasted with the use of MURA-based coded apertures, whose exact position and geometry are critical in the reconstruction process (Soltau *et al.*, 2023 ▸). Therefore, the present study aimed to perform hyperspectral XANES mapping analysis using a full-field XRF imaging method as a first feasibility demonstration, and show that such an approach can be potentially easily achieved when a FZP is employed as a coded aperture. This spectroscopic method has potential applicability in various research fields to determine 2D spatial distributions of the oxidation states of an X-ray absorber present at low concentration in samples, including beam-sensitive ones. Accordingly, a secondary goal of this study was to provide an optimized version of the CTF-based reconstruction algorithm. It will allow this easy-to-implement imaging technique to be readily employed at any research facility equipped with an FZP and an energy-resolved 2D detector, as well as lay a basis for future improvements and extensions of the reconstruction approach.

## 2. Material and methods

### 2.1. Principles of the reconstruction method

The basic principle of the image reconstruction method employed in this study has been detailed elsewhere (Soltau *et al.*, 2023 ▸). Its corresponding algorithm is derived from the one employed to reconstruct, via a contrast transfer function, holographic images (Lohse *et al.*, 2020 ▸). Briefly, if *S* corresponds to a radiation signal Ψ that has been successively emitted by an object, passed through an FZP used as a coded aperture, and recorded by a 2D energy-resolved detector, it can be employed to reconstruct Ψ following the relationship
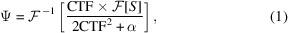
where 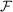 and 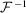 represent Fourier transform and reverse Fourier transform operators, respectively; α corresponds to a regularization function that avoids the denominator in the equation above to be equal to zero at *k* = 0 (Salditt *et al.*, 2017 ▸; Bartels, 2013 ▸) and improves the quality and stability of the phase retrieval (Zabler *et al.*, 2005 ▸); and the contrast transfer function is expressed as
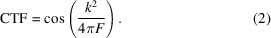
In the above equation, *k* is a grid of Fourier frequencies corresponding to the sampling points, and *F* is the Fresnel number expressed as
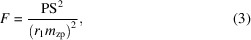
where PS is the pixel size of the bidimensional detector, *r*_1_ is the innermost zone radius of the FZP, and *m*_zp_ is the geometric magnification term, which is equal to the distance between the sample and detector (*z*_2_) divided by the distance between the sample and FZP (*z*_1_). Therefore, this method is dependent on just four experimental parameters, besides the signal *S* recorded by the detector. Two of these parameters are intrinsic to the experimental setup employed, *i.e.r*_1_ and PS, thus their values are constant. The values of the two other parameters, *i.e.z*_1_ and *z*_2_, are user-defined to achieve a given object magnification at the sensor position (Soltau *et al.*, 2023 ▸). From a practical standpoint, this method is very convenient since accurate knowledge of the values of *z*_1_ and *z*_2_ is not compulsory to achieve successful reconstruction. Indeed, as demonstrated in the *Results*[Sec sec3] section, the accurate values of *z*_1_ and *z*_2_ allowing optimal reconstruction can be empirically determined during post-data treatment.

### 2.2. Sample description

For testing the imaging approach employed in this study, a 2.54 cm × 2.54 cm, 0.15 cm-thick positive 1951USAF Resolution Test Target was employed (R1L17P, Thorlabs). It had a substrate made of clear soda lime glass and pattern design made of Cr, which was deposited onto the substrate surface via photolithography and corresponded to Groups 0–7 of the 1951USAF chart.

For testing the hyperspectral XANES mapping method, 0.1 *M* Cr(III) and 0.1 *M* Cr(VI) solutions were prepared by dissolving 1 g of chromium nitrate nonahydrate [Cr(NO_3_)_3_·9H_2_O] and 0.49 g of potassium chromate (K_2_CrO_4_) in 25 ml of ultra-pure deionized water, respectively. Both chromium solids were reagent grade (Sigma-Aldrich). Additionally, a specific volume fraction of the Cr(VI) solution was mixed with two equivalent volume fractions of the Cr(III) solution. Similarly, a specific volume fraction of the Cr(III) solution was mixed with two equivalent volume fractions of the Cr(VI) solution. Therefore, four solutions were prepared in total: the first, second, third, and fourth solution corresponded to 100% Cr(III) and 0% Cr(VI), 0% Cr(III) and 100% Cr(VI), 66% Cr(III) and 33% Cr(VI), and 33% Cr(III) and 66% Cr(VI), respectively. Four polyamide filter membranes (NL 17, Whatman), with 25 mm diameter, 110 µm thickness and 0.45 µm pore size, were individually dipped for about 10 s in a specific solution among the four chromium solutions prepared. The four membranes were then dried at room temperature for a few hours, cut to a quarter circle shape, and finally all placed together as a full circle shape while glued on top of a Kapton tape, which was itself attached, at its two extremities, to a standard slide mount (Reflecta) (Fig. S17 of the supporting information).

To determine how much Cr was retained on a membrane dipped in the solution containing only Cr(III) or Cr(VI), a filter membrane was dipped for about 10 s in the 100% Cr(III) solution, and another filter was similarly dipped in the 100% Cr(VI) solution. The membranes were then dried at room temperature for more than 48 h, dissolved in acid, and analyzed for chromium concentration by inductively coupled plasma optical emission spectroscopy (ICP-OES) using an ICAP Pro Series spectrometer (ThermoFisher), by Filab Laboratory (Saint-Apollinaire, France). This whole procedure was repeated five times, so that the amount of Cr(III) or Cr(VI) retained on the membrane, and its associated experimental uncertainty (*i.e.* standard deviation around the mean), could be determined from six replicates.

### 2.3. Experimental setup

**Figure 1:**
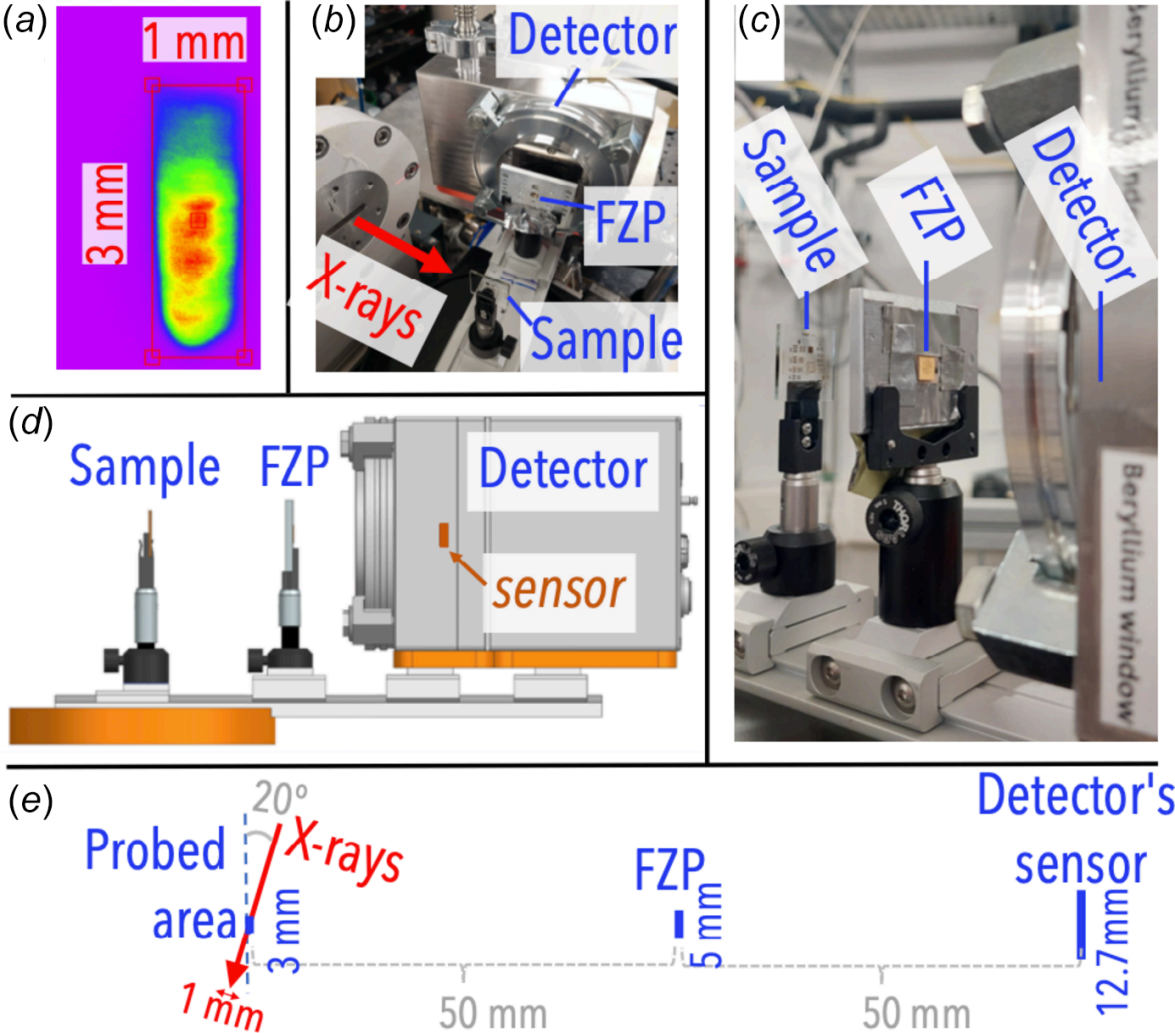
Experimental setup: (*a*) image at sample position of the 1 mm × 3 mm unslitted monochromatic X-ray beam at 5980 eV; (*b*) picture of the setup, high angle view; (*c*) picture of setup, profile angle view; (*d*) setup in model space, profile view – the sensor position inside the detector is indicated by an arrow; (*e*) schematic representation of the setup, top view. The beam path of the collected X-rays fluorescence is represented in pale green.

The experiments were performed at the SAMBA (Spectroscopy Applied to Materials Based on Absorption) beamline at Synchrotron SOLEIL, France. The machine was operated with a 450 mA current. A monochromatic beam was delivered by a Si(220) monochromator, which was calibrated at 5989 eV at the first inflection point of the XANES spectrum corresponding to a chromium (Cr) foil. The beam flux at 6000 eV was about 5.5 × 10^11^ photons s^−1^. The beam energy was first fixed at 6058 eV to collect images of the 1951USAF resolution target. It was then varied from 5980 to 6030 eV in order to collect XANES spectra at the chromium (Cr) *K*-edge. The X-ray beam was defocalized so that its size, measured using a Scout und Pioneer camera (Basler AG) mounted with a 12 mm TV lens (Pentax), which was placed at sample position at the perpendicular plane with respect to the direction of beam path, was 1 mm horizontally and 3 mm vertically [Fig. 1 ▸(*a*)]. The sample was tilted at a given angle with respect to the direction of the beam path, *i.e.* 20 and 15° to image the 1951USAF resolution target and for the hyperspectral XANES mapping trial, respectively [Fig. 1 ▸(*e*)]. The area probed by the beam on the sample was then 3 mm × 3 mm and 4 mm × 3 mm (H × V) in the former and latter cases, respectively.

The FZP apparatus (consisting of an FZP, outermask, and holder) was made by XRnanotech, Switzerland. The FZP had a gold (Au) contrast material, 5000 µm diameter, 25 µm outermost zone length, and 20 ± 2 µm thickness. Therefore, its innermost zone radius *r*_1_ was 0.354 mm, given the direct relationship between this parameter and the FZP’s diameter and outermost zone width (Wu *et al.*, 2020 ▸). The FZP was held by a 1 µm-thick silicon nitride membrane, surrounded by a 7 mm × 7 mm square-shape gold outermask, and finally glued on top of a 6 mm-diameter hole that was drilled in the center of a 4 mm-thick holder plate made of stainless steel. After reception of the FZP apparatus, its surface outside of the FZP area was covered with lead tape [Fig. 1 ▸(*c*)].

The bidimensional detector was an energy-resolved CXC camera (PnDetector GmbH, Germany). This camera is a single-photon-detection device, enabling the position, time, and energy of each photon reaching the detector to be recorded (Ordavo *et al.*, 2011 ▸). The detector sensor featured 264 × 264 pixels with a 48 µm effective pixel size, thus its size was 1.27 cm × 1.27 cm. It was operated at −20°C using a water-cooled Peltier system. The detector was under primary vacuum to ensure thermal insulation, and sealed using a 50 µm-thick beryllium window, which allowed the detector to be conveniently handled at ambient pressure.

The sample (*i.e.* the resolution target or the slide mount with filter membrane chunks attached to it) and FZP apparatus were individually held using standard optical post holders (Thorlabs, USA), while the CXC camera was held using a home-made 3D-printed holder. These three holders were placed on top of an optical construction rail (XT 34, Thorlabs, USA) via standard individual mounting platforms (Thorlabs, USA), which were screwed to the rail. The center of the sample, the FZP, and detector sensor were aligned along the rail center, and their respective surfaces were parallel to each other [Figs. 1 ▸(*d*) and 1(*e*)]. The rail itself was screwed, using a home-made 3D-printed adaptor, to the sample stage of the beamline experimental table, which was motorized in rotation and translation, both vertically and horizontally on the axis perpendicular to beam path. The rotation motor, whose rotation axis was aligned with the sample’s vertical holder post, was used to set the sample surface at a given angle with respect to the beam path direction [Fig. 1 ▸(*e*)]. The translation motors were employed to fine-adjust the vertical and horizontal positions of the beam onto the sample. Different sample–sensor and sample–FZP distances were employed in this study to achieve different image magnifications, as detailed in the *Results*[Sec sec3] section.

### 2.4. Image processing

The CXC camera continuously acquired image frames at a rate of 400 frames s^−1^ (2.5 ms per frame). Each frame consisted of a 264 × 264 pixel array. For each pixel, detected photon events were accumulated into an energy spectrum comprising 2000 energy bins spanning from 10 to 20000 eV with a bin width of 10 eV. During acquisition, the energy spectra corresponding to each pixel were progressively summed frame by frame over the full acquisition period, which typically lasted several minutes. The final dataset therefore consisted of a single three-dimensional data cube with dimensions (*x*, *y*, *E*), where each pixel position (*x*, *y*) was associated with a summed energy spectrum representing the total number of detected photons as a function of energy over the entire acquisition time. In this cube, the bins whose corresponding energies were within 5160–5600 eV (*i.e.* the energy range of the fluorescence emission of chromium) were summed together, and the resulting single [264 × 264] array, referred to as the ‘reconstruction input array’ (RIA) in this manuscript and the associated supporting information document (hereafter SI), was finally processed via the algorithm described below to reconstruct the image.

The reconstruction approach previously described in the *Methods*[Sec sec2] section was coded in a Matlab script featured in the *HoloTomo* toolbox (Lohse *et al.*, 2020 ▸). A shortened and modified version of this script was employed in this study. A version written in Matlab and an equivalent version transcribed to Python language are fully provided in SI. The expression of the CTF in the original Matlab code, *i.e.* CTF = 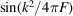, was modified so that it could correspond to the one shown above in equation (2), in accordance with the CTF expression defined in a previous study (Soltau *et al.*, 2023 ▸). Additionally, the code was modified in this study to account for the spatial resolution that may be virtually decreased because of the image reconstruction process, as further discussed in the *Results*[Sec sec3] section and SI.

### 2.5. XANES analysis

A hyperspectral XANES mapping measurement, performed at a specific absorption edge, consisted of acquiring an RIA at each energy of the XANES spectrum. Since the measurements were carried out around the Cr *K*-edge (5989 eV), the energy of the monochromatic X-ray beam delivered by SAMBA’s monochromator was set to 5980 eV, which corresponded to the lowest energy of the XANES, and below the energy corresponding to the Cr *K*-edge where the Cr *K*α fluorescence emission arises. As the sample was exposed to the X-ray beam at this specific energy, a single RIA was acquired by collecting 72000 frames successively acquired for 3 min by the CXC camera. Then, the energy of the incident X-ray beam was increased by 1 eV, thus 5981 eV, and an RIA was again obtained using the same approach as the one previously described. This procedure was successively repeated by gradually increasing the energy of the X-ray monochromatic beam by 1, 0.5, 1 and 2 eV in the energy ranges of the XANES corresponding to 5980–5989, 5990–5996, 5997–6013, and 6014–6058 eV, respectively. Therefore, a total of 65 reconstructed images were finally obtained, which could be grouped in a single [264 × 264 × 65] data array, or 69696 single [1 × 65] data arrays. All these single data arrays were processed using a home-made algorithm, to extract, for each pixel of the reconstructed sample object, the corresponding XANES spectrum. Details of all data processing steps in this code are described in SI. Once this XANES extraction step was completed, the data array corresponding to each XANES spectrum was saved as a text file. The text file featured two data columns, which corresponded to the specific incident X-ray energies and their associated intensities in the first and second column, respectively. This allowed all individual XANES spectra to be loaded into the XAFS data treatment software *Fastosh* (Landrot & Fonda, 2025 ▸). The raw, unnormalized XANES dataset was treated in *Fastosh* via a multivariate curve resolution alternating least-square (MCR-ALS) approach using a customized version of Jaumot *et al.*’s MCR-ALS toolbox (Jaumot *et al.*, 2015 ▸). This was done to extract the XANES spectrum corresponding to each pure chemical species constituting the sample mixture and determine their respective 2D distribution. During the alternating least-square refinement step, a non-negativity constraint was applied to both the coefficient and spectra matrixes, and a closure constraint was employed to the coefficient matrix. Therefore, the sum of the relative contents of the two pure species, obtained by MCR-ALS, was equal to 1 at each pixel location. The mean of these values, for each pure species, was calculated in each of the four sample regions corresponding to a given filter membrane chunk. This average was then compared with the real relative amount of Cr(III) or Cr(VI) present in each of the four sample regions, to determine the measurement’s accuracy. Additionnally, the standard deviation around the mean in the relative content values was also calculated for each pure species, and in each of the four sample regions, to assess the measurement’s precision.

## 3. Results

Once the experimental setup, consisting of a 1951USAF resolution target (Cr-containing pattern on glass), FZP, and CXC, was installed inside SAMBA’s experimental hutch (Fig. 1 ▸), multiple raw images at different sample locations were successfully collected at energies above the Cr *K*-edge. Several FZP and detector distances relative to the sample position were employed to test different image magnifications (Fig. S1).

**Figure 2:**
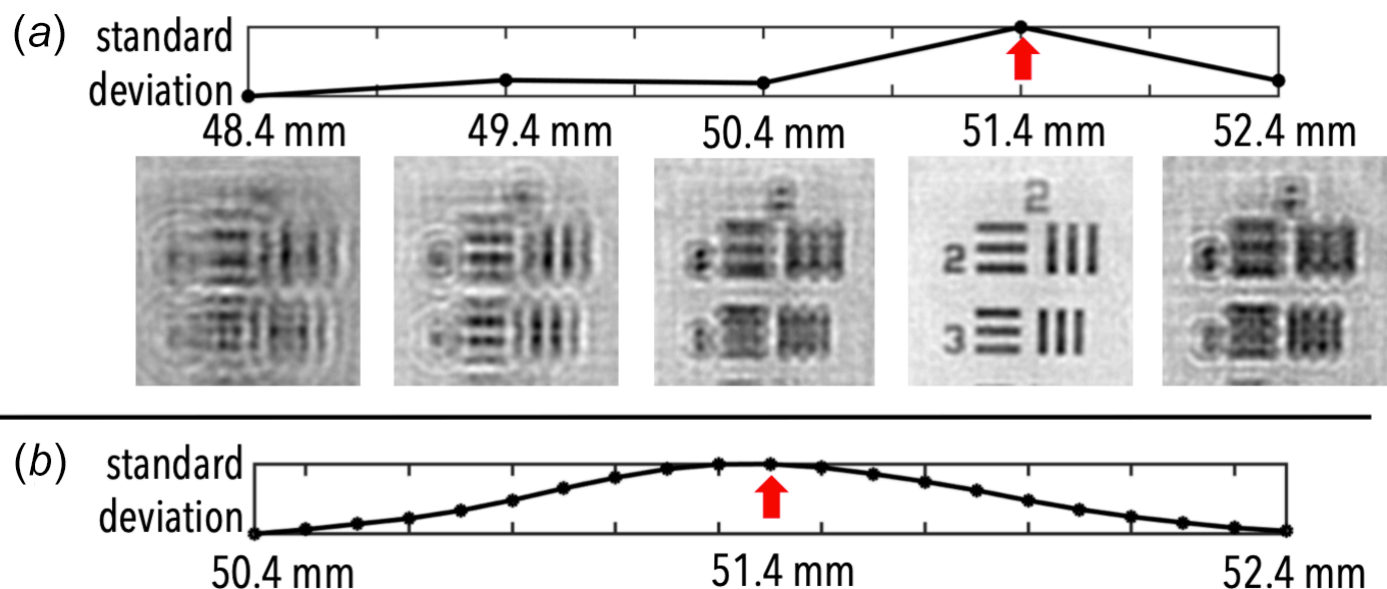
Standard deviation of the reconstructed image when the sample–FZP distance, which was experimentally measured as 50.4 mm, was incrementally modified in post-acquisition prior to performing the reconstruction, (*a*) firstly from 48.4 to 52.4 mm using a coarse 1 mm step (the reconstructed image obtained for each distance value is also shown), and then (*b*) from 50.4 to 52.4 mm using a fine 0.1 mm step. In both cases, the optimum distance value that provided the highest image standard deviation (*i.e.* 51.4 mm) is indicated by a red arrow.

The patterns of the resolution target featured in the reconstructed image could appear blurry when the two experimental distances, sample–FZP and sample–detector, which were set and measured by hand using a ruler inside the beamline’s hutch, were directly used as input parameters in the reconstruction algorithm. In this case, the reconstruction was repeated to improve the image quality, using again the two experimental distance values as input but with one of them being slightly refined by typically less than a few millimetres. Only one of these two distance values could be refined since it is actually their ratio that is employed as input parameter in the reconstruction algorithm, as shown in the *Methods*[Sec sec2] section. The quality of the sample image could be readily improved when the distances were empirically refined from visual observations (not shown). Alternatively, measuring the sum of the standard deviation of the reconstructed image, along both row and column pixel directions, could allow the best distance value providing optimum image quality to be found (Fig. 2 ▸). This method may be less effective, however, for unknown samples, especially those that are heterogeneous or with low contrast. Therefore, a future study should aim at determining the best distance-refinement approach that could be efficiently applied for any types of samples. Such an approach could be then included, as a fully automatized step, in the reconstruction algorithm supplied in SI.

**Figure 3:**
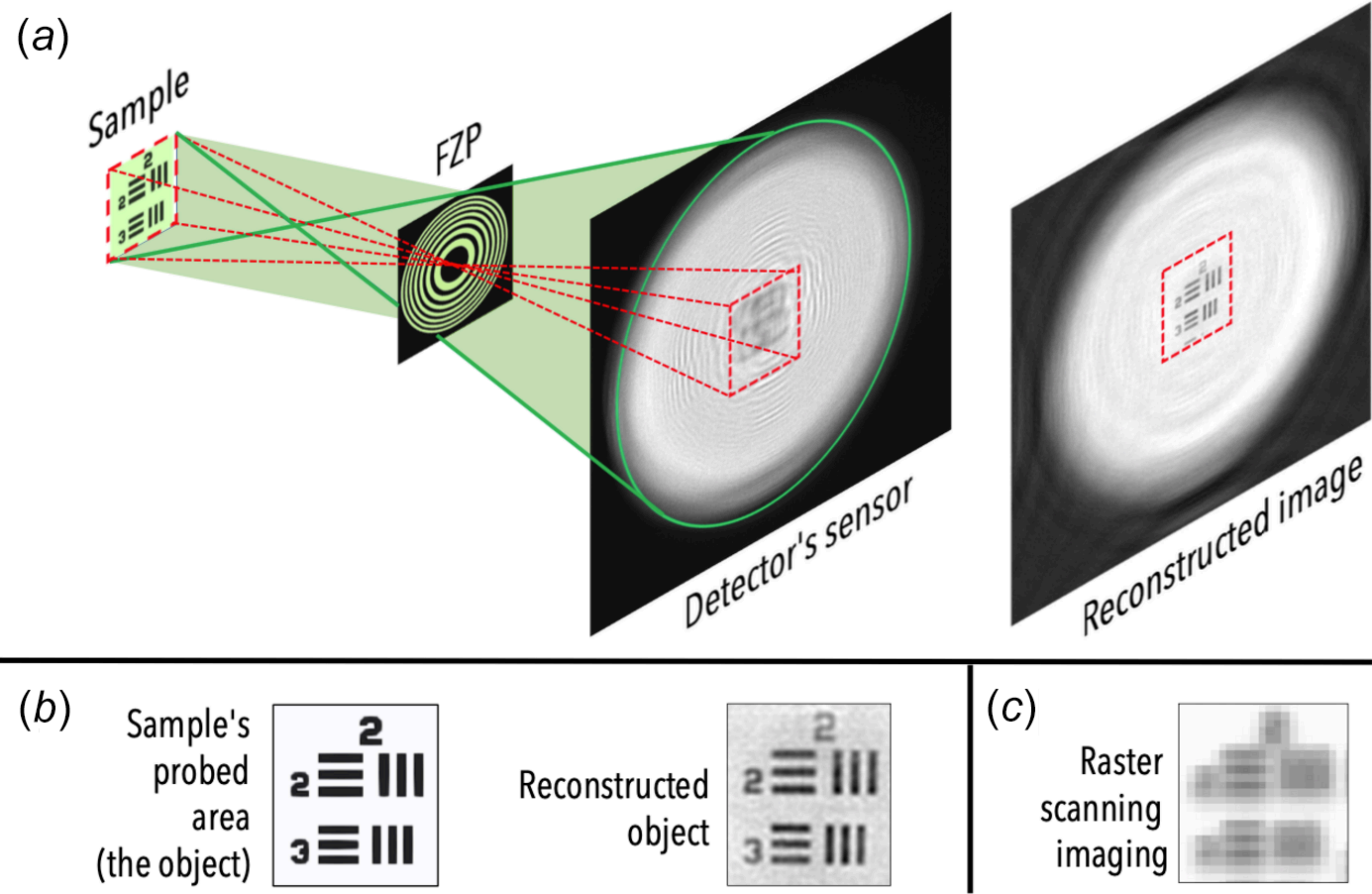
(*a*) Schematic representation of the setup illustrating the size difference, on the detector sensor, between the recorded raw signal, delimited by a green color circle, versus the reconstructed object (a region of the 1951USAF pattern) obtained after image reconstruction, delimited by a red dashed square. (*b*) Model image of a region featured in the 1951USAF sample and its corresponding reconstructed image experimentally obtained using the full-field imaging approach; and (*c*) the same object imaged in raster scanning mode using SAMBA beamline’s fully focused macroscopic beam (120 mm × 150 µm, H × V).

Once the optimum distance was determined, the sample object, featured in the reconstructed image whose corresponding raw image was collected using the full-field imaging approach in about 5 min [Figs. 3 ▸(*a*) and 3(*b*)], could be recognized much more clearly compared with the same sample object featured in an image collected in more than an hour at the same sample area using a traditional raster scanning mode and the fully focused X-ray beam of SAMBA [Fig. 3 ▸(*c*)].

The size of the light halo at the sensor plane, forming the raw image, depends on the size of the diameter of the FZP [Fig. 3 ▸(*a*) and Fig. S2]. In contrast, the size of the object featured in the reconstructed image follows the pinhole model (Wu *et al.*, 2020 ▸); it is thus not dependent on any inherent physical characteristics of the FZP [Fig. 3 ▸(*a*) and Fig. S2]. Consequently, the number of pixels carrying the object information decreased between the raw and reconstructed images, *i.e.* ∼260 versus 58 pixels, respectively. The basic form of the reconstruction algorithm was then modified to compensate for this decrease, as further detailed in SI.

**Figure 4:**
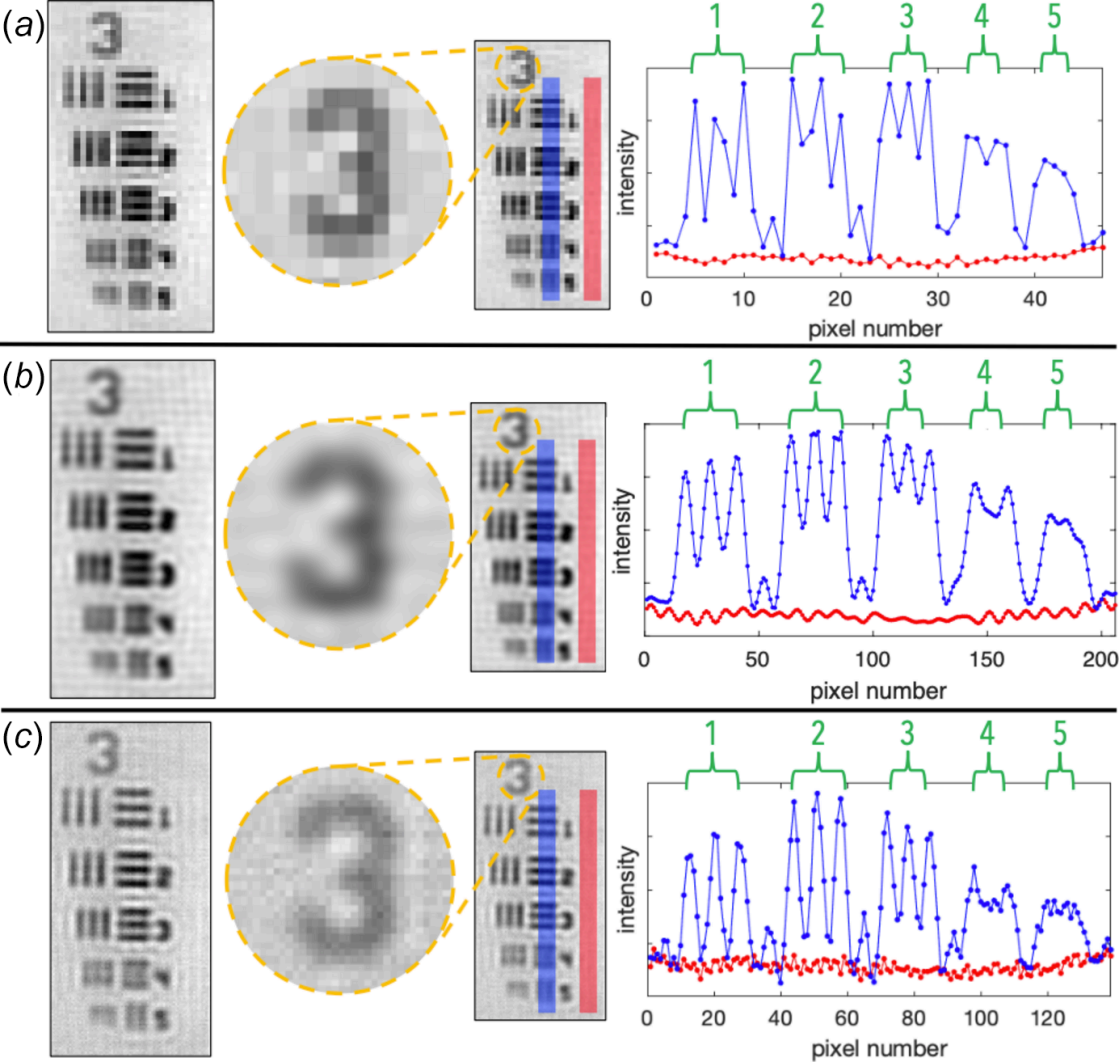
Reconstructed image of Elements 1–5 belonging to Group 3 of the 1951USAF pattern, and the sum in pixel intensities measured in this image at locations corresponding to a bar, colored in blue or red color and passing across Elements 1–5 (labeled in green color) or the background, respectively, when (*a*) no data processing was performed prior to reconstruction; (*b*) the size of the raw image was enlarged using the resizing factor (see text) prior to reconstruction; and (*c*) a subpixel analysis approach was performed prior to reconstruction using a 16 µm virtual pixel size. All the raw images corresponding to the reconstructed images shown in this figure were collected under identical conditions (*i.e.* same distances and acquisition time period).

With our modified reconstruction algorithm, the spatial resolution of the reconstructed image [Fig. 4 ▸(*b*)] was obviously much better than when this operation was not performed prior to reconstruction [Fig. 4 ▸(*a*)], *i.e.* when the basic reconstruction method described in the *Methods*[Sec sec2] section was used. The resolution of the image could be further improved using a data treatment called subpixel analysis (Nowak *et al.*, 2015 ▸), where the centroid of the charge cloud generated by the absorption of the photon in the sensor could be determined more accurately by virtually employing a smaller pixel size than the original one of the detector, at the cost of decreasing the image signal-to-noise ratio [Fig. 4 ▸(*c*)]. The experimental results obtained using the three data treatments, shown in Fig. 4 ▸, which are further discussed and compared with each other in SI, were consistent with those obtained using a direct ray-tracing approach and reported in SI.

The widths of the bars, and the spaces between them, featured in Elements 1, 2, 3, 4, and 5 of Group 3 of the 1951USAF resolution target are 62.5, 55.6, 49.6, 44.2, and 39.4 µm, respectively. When the raw image was resized prior to reconstruction, the 62.5 µm-wide spaces between the bars featured in Group 3’s Element 1 could be distinguished but were not entirely resolved as their corresponding pixel intensities did not reach background level [Fig. 4 ▸(*b*)]. It could be then reasonably assumed that the spatial resolution at the object plane, visually assessed from direct observations of the resolution target reconstructed image, was above, but not too far from 62.5 µm. In contrast, these spaces featured in Element 1 were just entirely resolved in the image derived from sub-pixel analysis as their corresponding pixel intensities barely reached background level, whereas the 55.6 µm-wide spaces between the bars of Group 3’s Element 2 were not entirely resolved. Therefore, in this case, the spatial resolution at the object plane seemed to be around 62.5 µm.

**Figure 5:**
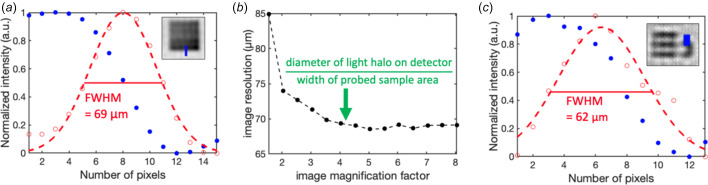
Spatial resolution at the sensor position measured in a reconstructed image obtained: (*a*) after enlarging its corresponding raw image using the resizing factor; or (*c*) from subpixel analysis. It was determined from the full width at half-maximum (FWHM) of a Gaussian curve fitted to the column pixels corresponding to the interface between a sample feature and the background, shown in the top right insets as a blue-colored bar. (*b*) The spatial resolution was also measured in a reconstructed image whose corresponding raw image was resized using multiple enlargement factors of increasing values. Among them, the specific value corresponding to the resizing factor introduced in this study is indicated by a green arrow.

The spatial resolution at the sensor plane was determined via a conventional approach, *i.e.* the step-edge method (Fig. 5 ▸). It was found to be 69 µm and 62 µm in the image whose corresponding raw data was resized prior to reconstruction using the resizing factor [Fig. 5 ▸(*a*)] and obtained from subpixel analysis [Fig. 5 ▸(*c*)], respectively. These results were thus similar to the spatial resolution at the object plane inferred from direct observation of the resolution target (Fig. 4 ▸). When the resizing approach was employed prior to reconstruction, the enlargement value corresponding to the resizing factor represented the optimum value for maximizing the spatial resolution at the sensor plane (*i.e.* the optimization criterion), since enlarging the raw image by a factor above this value did not improve the spatial resolution, which reached a plateau at around 70 µm [Fig. 5 ▸(*b*)]. While the 62 µm spatial resolution was obtained from an image derived from subpixel analysis using a 9.5 µm virtual pixel size [Fig. 5 ▸(*c*)], a similar spatial resolution (*i.e.* 63 µm) was obtained with a 16 µm virtual pixel size (Fig. S4). However, the spatial resolution was progressively degraded when decreasing the virtual pixel size at 6.8 µm or below this value, presumably due to a reduction in event statistics per virtual pixel as physical hits are distributed over a larger number of sub-pixels (Fig. S4). Therefore, the optimum and most reliable experimental spatial resolution at the sensor plane measured in our system was 62 µm. It was obtained when the raw image was generated with the subpixel analysis approach, using a 9.5 µm-virtual pixel size. The corresponding spatial resolution at the object plane should be close to 62 µm, since the FZP was positioned about half way between the sample and detector. This experimental spatial resolution, and the one inferred at the object plane from direct observation of the resolution target image (around 62.5 µm), were similar to those theoretically expected. Indeed, as shown in SI, the theoretical spatial resolution at the sensor and object planes were 59.3 µm and 62.6 µm, respectively.

**Figure 6:**
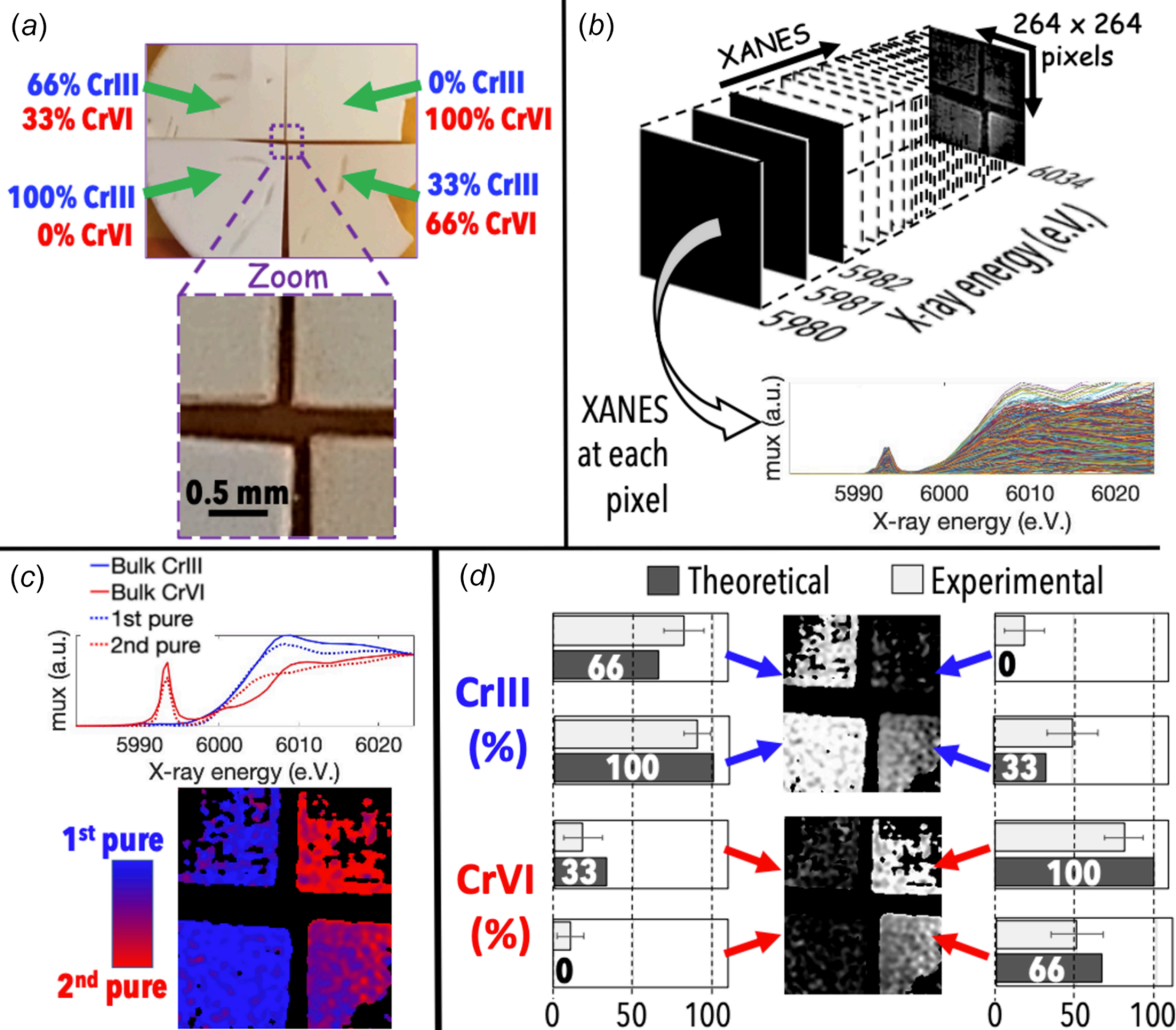
(*a*) Sample employed for the hyperspectral XANES mapping and, inside it, field of view probed by the X-ray beam. (*b*) Schematic representation of the method employed. (*c*) Spatial distributions and XANES spectra, extracted by MCR-ALS, corresponding to the two pure species present in the sample, which are compared with the XANES spectra corresponding to the individual membrane chunck exposed to the 100% Cr(III) or Cr(VI) solution, collected at the bulk scale and at the end of the hyperspectral experiment. (*d*) Relative amounts of Cr(III) and Cr(VI) present in the sample estimated using this technique, with associated error bars corresponding to the standard deviation around the mean. These values should be considered preliminary, as the quantification depends on empirical intensity-correction steps that have not been independently validated.

After commissioning the imaging reconstruction method on  the 1951USAF target, we applied XRF–XANES hyper­spectral analyses to Cr-bearing membranes. Given the Cr concentrations measured in these samples, fluorescence mode was more suitable than transmission mode to analyze these samples, as detailed in SI. The control XANES spectra, corresponding to the membrane chunks that were initially dipped into the 100% Cr(III) or Cr(VI) solution, and individually analyzed at completion of the hyperspectral mapping experiment and at the bulk scale using a regular XRF detector, were consistent with a pure Cr(III) or Cr(VI) chemical form [Fig. 6 ▸(*c*)]. Therefore, no significant change in Cr oxidation states occurred during the course of the experiment, *i.e.* from the step where the Cr(III) and Cr(VI) solutions were prepared to the step where the Cr-bearing membranes were imaged.

The XANES spectra corresponding to the two main ‘pure’ species, extracted by MCR-ALS from the dataset collected via the hyperspectral approach, were similar but not identical to the corresponding control XANES spectra [Fig. 6 ▸(*c*)]. Likewise, although the spatial distributions of these two species [Fig. 6 ▸(*c*)] were consistent with the expected distributions of Cr(III) and Cr(VI) in the sample [Fig. 6 ▸(*a*)], their relative amounts, experimentally measured by hyperspectral XANES mapping in each of the four membrane chunks, were off from the expected values by about 20% [Fig. 6 ▸(*c*)]. As detailed in SI, we attributed the main source of error in extraction of the XANES spectra corresponding to pure Cr(III) and Cr(VI) to the imperfect approaches employed to rectify the pixel intensities in the raw data, which were affected by the non-linear response of the detector. These approaches also affected the quantification results, but to a minor extent. Indeed, results obtained from theoretical hyperspectral XANES mapping performed using a direct ray-tracing approach suggested that, even if the sensor response was perfectly linear, the method’s accuracy and precision would still be off, due to the inherent nature of the reconstruction approach, by about 15 and 10%, respectively (Fig. S15 and Fig. S16).

## 4. Discussion

Based on the theoretical expressions of the spatial resolution at the object and sensor planes, which are provided in SI, the best spatial resolution experimentally achieved in this study at the object plane (*i.e.* around 62 µm) was very close to the theoretical value (59 µm). This resolution could be potentially improved by modifying the nature of the FZP and/or detector, while keeping the same field of view as the one employed in this study (*i.e.* 3 mm × 3 mm), and the same sample–detector distance (*i.e.* 10 cm). Firstly, using our same experimental setup, but with a smaller FZP, featuring a 1 mm diameter and 10.1 µm outermost zone width, and a sample–FZP distance at 2.55 mm, the spatial resolution at the object and sensor planes would be 16.5 and 48.3 µm, respectively (Fig. S9A). In this case, the spatial resolution would be still limited by the FZP outermost zone width, since *r*_sensor_ would remain higher than the CXC’s pixel size as was also the case in this study (*r*_sensor_ = 59.3 µm). Similarly, the use of a larger sensor area than the one employed in this study (*i.e.* 12.7 mm per square edge) could also result in an improved spatial resolution compared with the one we achieved, even with a pixel size larger than 48 µm. For instance, the very same FZP of this study combined with the 1 Mpixel version of the CXC camera (Kuster *et al.*, 2021 ▸), which features a 78 mm-large area and 75 µm pixel size, and positioned at 1 and 10 cm from the sample, respectively, could provide spatial resolutions at the object and sensor planes of 34 and 305 µm, respectively (Fig. S9B). Lastly, the use of an FZP featuring a 1 mm diameter, 4 µm outermost zone width, and 5 µm thickness, along with the 1 Mpixel version of the CXC camera, placed at 0.5 and 10 cm from the sample, respectively, could result in a spatial resolution at the object and sensor planes equal to 5.1 and 97.6 µm, respectively (Fig. S9C).

A given photon reaching the detector generates an electron cloud in the sensor, which may be smeared over multiple pixels. The photon energy is determined from the sum of the charges in these pixels (Ordavo *et al.*, 2011 ▸). Since one photon at 10 keV induces an electron cloud of about 100 µm in diameter in the sensor (Leitenberger *et al.*, 2008 ▸), one photon at 5.4 keV (*i.e.* Cr *K*α energy) should produce a cloud covering not more than 2 × 2 pixels, given the 48 µm pixel size. In this study, the processor of the CXC systematically employed a filter to generate the raw data only from events corresponding to a maximum of four pixels present within a 2 × 2 pixel area (Leitenberger *et al.*, 2008 ▸). Therefore, this filter allowed, during each frame-recording cycle, to detect each single Cr fluorescence photon reaching an isolated area of the sensor, and neglect groups of more than one Cr fluorescence photon reaching the detector with at least five neighboring pixels and resulting in a single, large electron cloud. This cloud, if not filtered, would be detected as a single photon with an energy higher than the one expected for Cr (*i.e.* pile-up). This amount of undetected Cr counts, corresponding to Cr photons that reached the detector at similar time and sensor locations, which represented at least between 5 and 15 adjacent pixels in the sensor as shown in Fig. S13, explains why the response of the detector was not linear during our spectroscopic trial. Therefore, employing a strategy that would account for these missing counts would certainly improve the extracted XANES spectra representative of pure Cr(III) and Cr(VI) reported in Fig. 6 ▸. It could consist of deactivating the CXC’s data-processing filter at data acquisition, so that all input counts would be accounted for, and subsequently using a pile-up peak-correction in post-data acquisition, to convert all Cr-corresponding pile-up counts to actual Cr counts with an energy around 5.4 keV, as shown in Fig. S14. It was also theoretically demonstrated that the main chemical species present in the sample could perfectly match those expected if the detector response was linear (Figs. S15 and S16). This was achieved without employing any masking, background treatment, and intensity-correction empirical steps prior to performing MCR-ALS (Fig. S17), as the pixel intensities of the object were well above those of the background.

Future work should aim at testing the experimental robustness and evaluating the sensitivity of extracted species maps to reasonable variations in the empirical steps employed in this study to rectify the effect of the non-linearity response of the detector, since their use may be necessary to study diluted specimens whose corresponding pixel intensities are similar to those of the background. Additionally, given that the present workflow was framed as a proof-of-concept demonstration rather than a validated quantitative speciation method, an independent validation, for example by micro­probe raster XRF-XANES measurement, would strengthen confidence in the reported quantitative results. Lastly, while the four-quadrant membrane assembly represented a reasonable internal reference for a feasibility study, and despite its chemical composition not appearing to vary during the spectroscopy trial based on the Cr(III) and Cr(VI) XANES spectra collected at the bulk scale and at the end of the hyperspectral experiment [Fig. 6 ▸(*c*)], our sample was fundamentally less reliable than an independently calibrated quantitative standard with known areal densities. Therefore, the latter should be preferentially employed to perform an independent validation.

## 5. Conclusions

In this study, full-field imaging in fluorescence mode, which could be easily achieved using an FZP as a coded aperture, was employed to image an object at a beamline not dedicated to X-ray imaging. The results could be obtained four times faster compared with when a traditional raster scanning imaging approach was employed at the same beamline, while the photon flux density on the probed sample area was 500 times lower. Moreover, the basic reconstruction method relying on the CTF function was successfully optimized with an algorithm that strikingly improved the quality of the reconstruction image. Additionally, some basic, crucial aspects of the technique were clarified, such as the expression of the spatial resolution, which was revised as detailed in SI. This knowledge, by itself, will efficiently help the widespread use of this imaging approach, notably to adequately choose the nature of the experimental setup (characteristics of the FZP and detector) to fit the users’ specific research needs. Indeed, considering the same field of view as the one achieved in this study (*i.e.* 3 mm × 3 mm), it was shown in this investigation that the spatial resolution may be potentially increased to less than 10 µm using an FZP and sensor smaller and larger than those employed in this study, respectively. This imaging method has potential applicability in various research fields to determine the 2D spatial distributions of different diluted elements, including those present in beam-sensitive samples.

The hyperspectral XANES mapping, corresponding to the spectroscopic variant of the full-field XRF imaging method, was performed for the first time as a feasibility trial. Further development of the data processing workflow and independent quantitative validation should be done to improve the reliability of this approach to map in 2D the oxidation states of chemical elements, including those present in samples at low concentrations. Fast worldwide development of detectors should also provide, at short terms, the tools to meet the needs for efficiently performing hyperspectral XANES mapping. Indeed, while the development of a future CXC with higher frame and count rates is currently ongoing (Ordavo *et al.*, 2011 ▸), an energy-resolved bidimensional detector with saturation count rates above 500 Mcps, 17.4 kHz frame rate, and featuring a 28 mm × 53 mm-large sensor area and 73 µm pixel size, was recently conceived (Miao, 2025 ▸).

## 6. Related literature

The following references, not cited in the main body of the paper, have been cited in the supporting information: Calvin (2013 ▸).

## Supplementary Material

Sections S1 to S5, Table S1, Figs S1 to S18, and Python and MATLAB reconstruction scripts. DOI: 10.1107/S1600577526004789/vy5048sup1.pdf

## Data Availability

The data that support the findings of this study are available in the data repository ‘Recherche Data Gouv’ with the identifier https://doi.org/10.57745/7ZXWAV.
