# Supplementary material for: Full-field X-ray fluorescence imaging using a Fresnel zone plate as a coded aperture: optimized reconstruction algorithm and first trial of hyperspectral XANES mapping
Source: J Synchrotron Radiat. 2026 Jun 1;33(Pt 4):1093–102. doi: 10.1107/S1600577526004789 (PMC13344536; doi:10.1107/S1600577526004789)
Supplement: Supplementary file 1 [file s-33-01093-sup1.pdf]

JOURNAL OF SYNCHROTRON RADIATION

Volume 33 (2026)

Supporting information for article:

Full-field X-ray fluorescence imaging using a Fresnel zone plate as a coded aperture: optimized reconstruction algorithm and first trial of hyperspectral XANES mapping

Gautier Landrot, Arkadiusz Dawiec, Björn Eckert, Petra Majewski, Michael Brunner, Andrew Fram, Martin Huth, Guillaume Alizon, Claude Menneglier, Enrico Piasentier, Karine Chaouchi, Stéphanie Blanchandin, Pascal Mercere, Martin Chauvin and Emiliano Fonda

Table S1 Comparison in setup approaches, energy and spatial resolutions relative to the full-field X-ray fluorescence technique tested at a few beamlines worldwide

Reference	Beamline	Experimental approach(es)	Detector	Energy-resolved	Energy resolution	Spatial resolution
Kulow et al. 2020	BAMline, BESSY II (HZB), Germany	polycapillary apparatus<br>Coded aperture: 12-pinhole MURA (rank 5)	PnCCD (PnDetector)	yes	145 eV at 6 keV	undisclosed (> 48 μm)<br>90 μm
Siddons et al. 2020	XFP beamline, NSLS-II, USA	Coded aperture: MURA (orders 19, 37, and 73)	Medipix detector	no	none	between 14.3 & 25 μm
Zhao et al. 2019	BL-14B, Photon Factory, Japan	Polycapillary apparatus	C4880-50 CCD (Hamamatsu)	yes	150 eV at Mn Kα	18 μm
Klysubub et al. 2023	BL8, SLRI, Thailand	Polycapillary apparatus	PnCCD (PnDetector)	yes	145 eV at 6 keV	68 μm

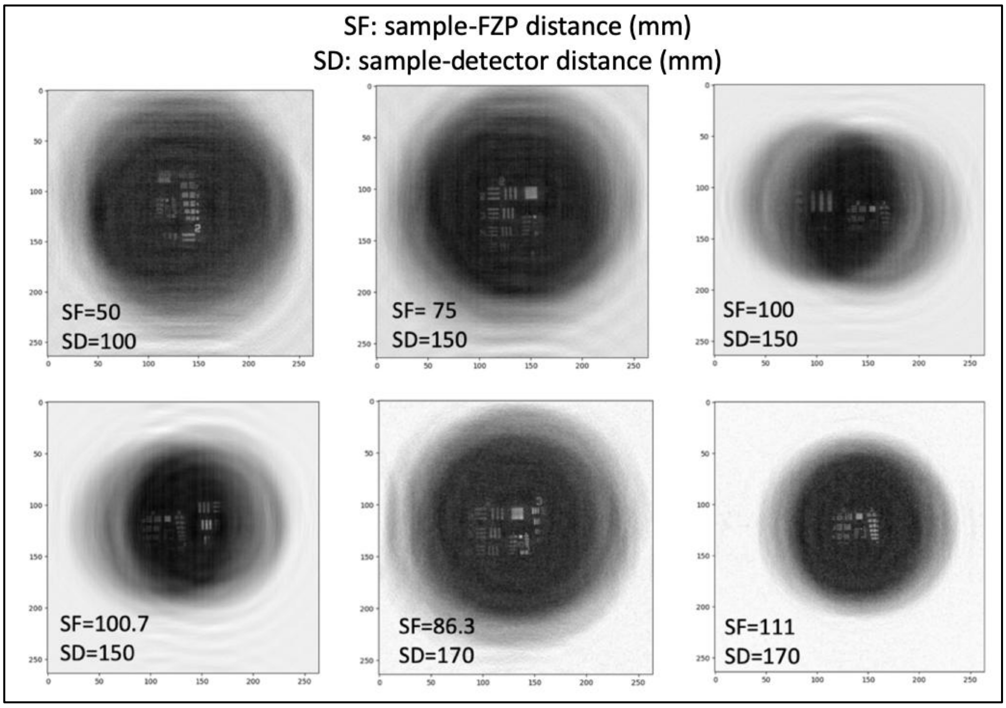

Figure S1 Images of the 1951USAF corresponding to different sample zones probed, with specific sample-detector and sample-Fresnel Zone Plate (FZP) distances, each of them collected in about 5 minutes.

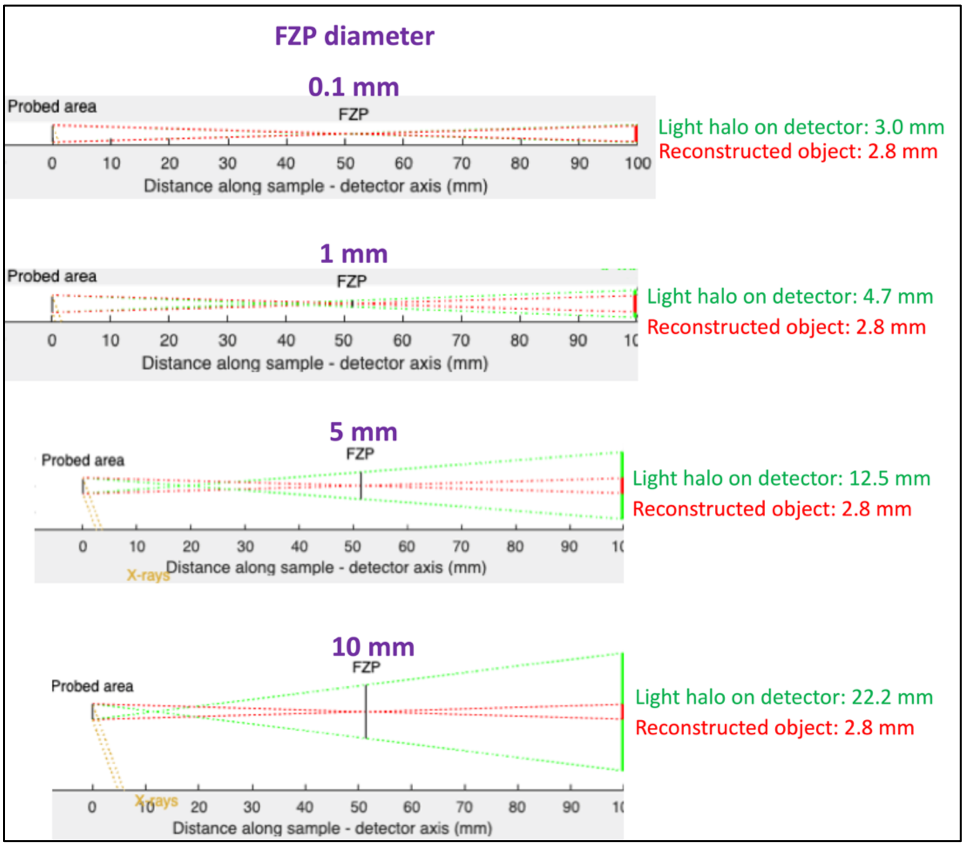

Figure S2 Sizes, at sensor position, of the light halo forming the raw image and the reconstructed object, for various FZP diameters and with a 3mm probed area.

S1. Results: main modification of the basic CTF-based reconstruction method

The size of the light halo at sensor plane, forming the raw image collected by the detector, depends on the size of the diameter of the FZP that is used as a mask of light, as schematized in Figure S2. The larger the FZP diameter, the bigger the ratio between the size of the light halo at sensor plane and size of the object featured in the reconstructed image (Figure S2). In contrast, since the size of the object featured in the reconstructed image follows the pinhole model (Wu *et al.*, 2020), this size is not dependent on any inherent physical characteristics of the FZP (Figure S2).

With a 5-mm diameter FZP employed in this study, the size of the light halo (12.5 mm), which covered most of the detector sensor's surface (12.7 mm), was more than four times larger than the size of the field of view (2.8 mm) inside the reconstructed image. The entire reconstructed image had the exact same pixel number and size as those of the raw image, since the pixel grid is conserved in the reconstruction algorithm (Fig. 3A). Therefore, while the light halo containing the object information covered about 260 pixels of the sensor in the raw image, the object that was reconstructed using the

method derived from inline holography and described in Methods only covered about 58 pixels in the reconstructed image, given that the pixel size was 48 µm. This decrease in number of pixels carrying the object information, from raw to reconstructed images, virtually decreased the spatial resolution. Since the raw and reconstructed images ideally carry the same object information, the spatial resolution should not be affected by the reconstruction procedure if pixel size was not a limiting factor. The reconstruction method described in Methods was thus modified to account for this aspect. In our modified algorithm, the size of the raw image is multiplied by the ratio between the size of the light halo forming the raw image and size of the field of view inside the reconstructed image, i.e. 4.5 (12.5/2.8) in the above example. Such approach requires an additional input parameter in the corresponding algorithm, in addition to those described in Methods, i.e. the length of the square edge of the field of view at sample location, which is assumed in the code to have a square shape. The value of this parameter is needed to calculate the sizes of the light halo forming the raw image & field of view in the reconstructed image to infer the factor employed to resize the raw image prior to reconstruction. An image comparison made from the same raw image resized prior to reconstruction, using different resizing factors, is shown in Figure S3. All raw or reconstructed images seemed identical, which suggested that the function used to resize the image (Matlab's imresize function, or its Python equivalent) did not create any noticeable artefact in the enlarged images as a consequence of interpolation, for any resizing factor employed (Figure S3).

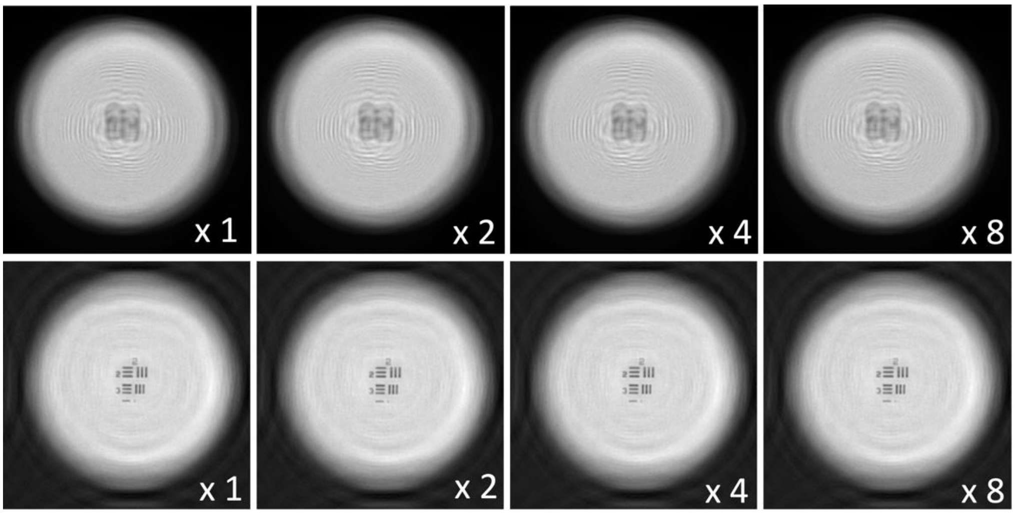

Figure S3 Raw (top row) and reconstructed (bottom row) images obtained with a specific image enlargement factor applied to a raw image prior reconstruction and displayed at the bottom right corner of each image. Although these images were eventually resized to the same dimensions so that their appearance could be conveniently compared to each other in this figure, their differences in pixel numbers were preserved (i.e. no image compression was applied), thus their final enlarged aspect was not modified in this figure.

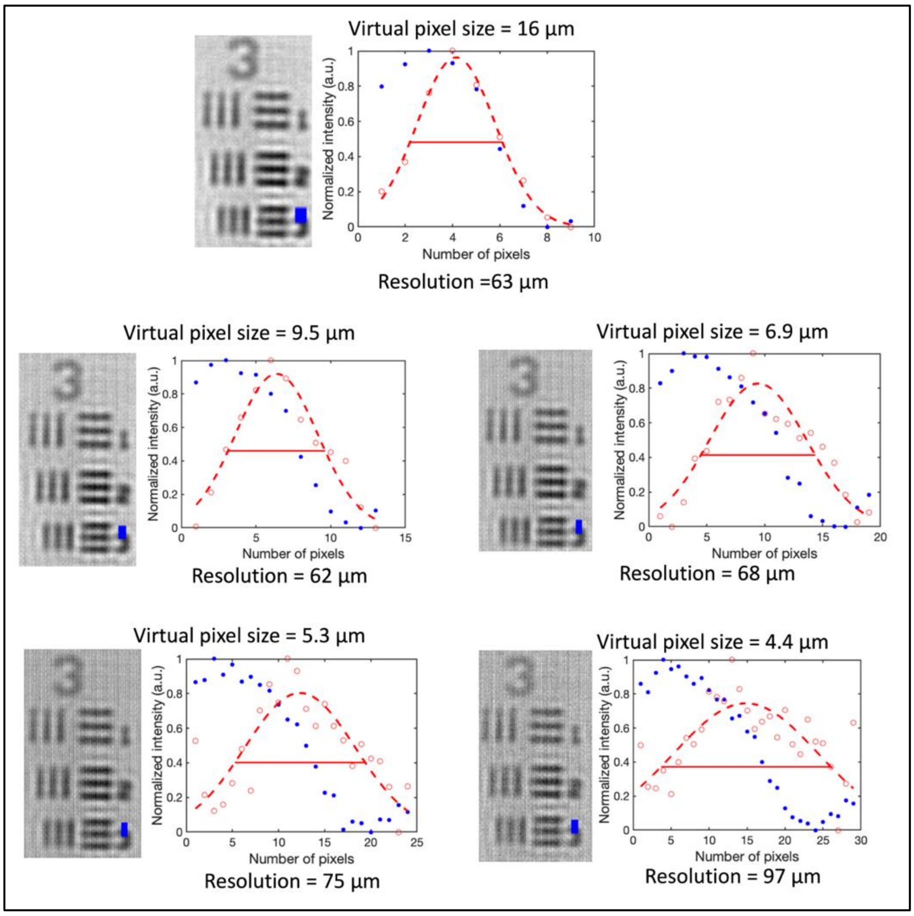

Figure S4 Spatial resolution at sensor plane measured, using the step-edge method, in a reconstructed image whose corresponding raw image was obtained from subpixel analysis using various virtual pixel sizes, from 16 to 4.4 µm.

S2. Results: comparison between experimental & theoretical spatial resolutions

For a system where a FZP is employed as a coded aperture, the spatial resolution of the probed object can be theoretically expressed as r = 1.22 * Δr, Δr being the width of the outermost zone, without considering the magnification (Wu *et al.*, 2020). If the effect of magnification is included in this expression, one must also include the divergence of the light originating from a sample, passing through a given zone of the FZP, and reaching the detector sensor where it contributes to the formation of the raw image. For example, the width of a light shaft originating from a source and passing through a given zone of a FZP, placed half way between the source and detector, is two times smaller at the FZP plane than at sensor plane, although the magnification factor M, defined as the ratio between the FZP-detector and sample-FZP distances, is in this case equal to 1. The spatial resolution experimentally measured in Wu *et al.* (2020) was found to systematically match the value of r, when multiple FZP of specific inner

radius were tested, although the sensor was placed very close to the FZP (1 % of the sample-detector distance). If the resolution at the *object plane* was expressed as r_{object}= r /M as in Wu *et al.* (2020) when only the effect of magnification is considered, and since M is also proportional to the ratio between the width of the reconstructed image at sensor plane and the width of the object at sample plane as mentioned in Wu *et al.* (2020), then the corresponding resolution at *sensor plane* would be, conversely, r_{sensor}= r * M. Therefore, to be consistent with the latter expression that only accounts for the magnification, the r_{sensor} experimentally measured in Wu *et al.* (2020) should have been actually much smaller than r, since the value of M was tiny given that the sensor was very close to the FZP. With the FZP-coded aperture system, considering both the magnification and divergence of a light passing through a virtual FZP hole of width r and reaching the sensor plane where the raw image is collected, the width of the projected hole on the sensor, equivalent to r_{sensor} , can be expressed, based on geometry (Figure S5), as:

$$r_{sensor} = r + r * M \qquad (4)$$

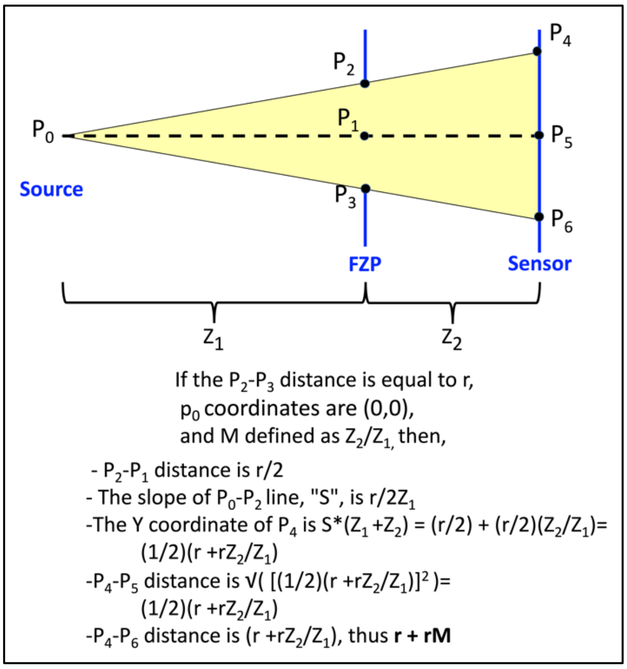

Figure S5 Schematic representation of a light, originating from a source, passing through a single FZP zone of width r, and reaching a detector sensor. From this sketch, the mathematical expression of the light width at sensor plane, schematized as the distance P_4-P_6, is defined.

Expression (4) agrees with the experimental resolution measured in Wu *et al.* (2020) since the tiny value of the M parameter in their experiments minimized the r * M term in the above equation, thus the resolution at sensor plane was indeed close to r. In case of our experiment, r was equal to 1.22 x 25 µm = 30.5 µm, and the theoretical resolution at sensor plane was then, based on Expression (4), r_{sensor} =

30.5 µm + 30.5 µm * (4.86 / 5.14 cm) = 59.3 µm. Therefore, the spatial resolution at sensor plane experimentally measured from the reconstructed image derived from subpixel analysis (62 µm, Figure 5 & Figure S4) and theoretical one (59.3 µm) were also similar to each other in our study. The spatial resolution at the object plane can be inferred from the one measured at sensor plane, considering the size ratio between the actual object and the one featured in the reconstructed image (i.e. M), $r_{object} = r_{sensor} / M$, or:

$$r_{object} = r + r \ / M \quad (5)$$

Based on the above expression, the theoretical resolution at object plane was in the present study r_{object} = 30.5 µm + 30.5 µm / (4.86 / 5.14 cm) = 62.6 µm. This value was thus consistent with the spatial resolution inferred from direct observation of the resolution target, using the reconstructed image derived from the subpixel analysis, where the 62.5 µm-wide spaces of Group 3's Element 1 seemed to be barely entirely resolved (Figure 4C).

S3. Results: Comparison between the three data-treatment methods shown in Figure 4

Reconstructing the raw image with our modified script led to a reconstructed image with a spatial resolution that appeared to be much better than the one obtained when the raw image was reconstructed right away with no enlargement. Although this latter spatial resolution could not be properly quantified using the step-edge method since the original pixel size did not allow to have enough pixels covering a given sample edge, it was above 85 µm, which corresponded to the spatial resolution measured when the raw image was resized by a factor of 1.5 (Figure 4B). Therefore, it was demonstrated that the modification made to the basic reconstruction method described in Methods, consisting in resizing the raw image prior reconstruction, represented an improvement to the reconstruction approach already available. Although the spatial resolution measured at sensor plane using this new approach was not as good as the one obtained when a subpixel analysis was performed to generate the raw image, it was close to it (i.e. 69 vs 62 µm). On the other hand, the signal-to-noise ratio of the reconstructed image obtained using the resizing method applied to the raw image was better than the one obtained using the subpixel analysis method (Figure 4B & C). This was expected since the raw images employed in both cases were acquired for the same time period, and the fact that the subpixel analysis approach is known to potentially increase spatial resolution at the cost of statistics, as the virtual reduction of pixel size essentially divides the number of counts in each pixel (Nowak *et al.*, 2015). Therefore, the subpixel analysis approach, compared to the image-enlargement approach, requires a longer acquisition time period to achieve the same image contrast.

Similar results were obtained when a direct ray-tracing approach was employed to generate a theoretical raw image corresponding to the 1951USAF Group 3 pattern (Figure S6). Indeed, based on

visual observations, the spatial resolution was increased when the theoretical raw image was enlarged prior reconstruction compared to when the basic reconstruction method was employed (Figure S7). Also, in agreement with the experimental results, the spatial resolution measured when the theoretical raw image was enlarged prior to reconstruction was a bit lower than the one obtained when the pixel size of the sensor was decreased (Figure S7). Therefore, given that it allowed to rapidly obtain a satisfying image contrast, and the fact that the optimal spatial resolution was not mandatory to perform hyperspectral X-ray Absorption Near-Edge Structure (XANES) mapping, all raw images obtained from the hyperspectral XANES mapping experiment were achieved using the data-treatment approach introduced in this study, consisting in virtually augmenting the size of the raw image prior to reconstruction.

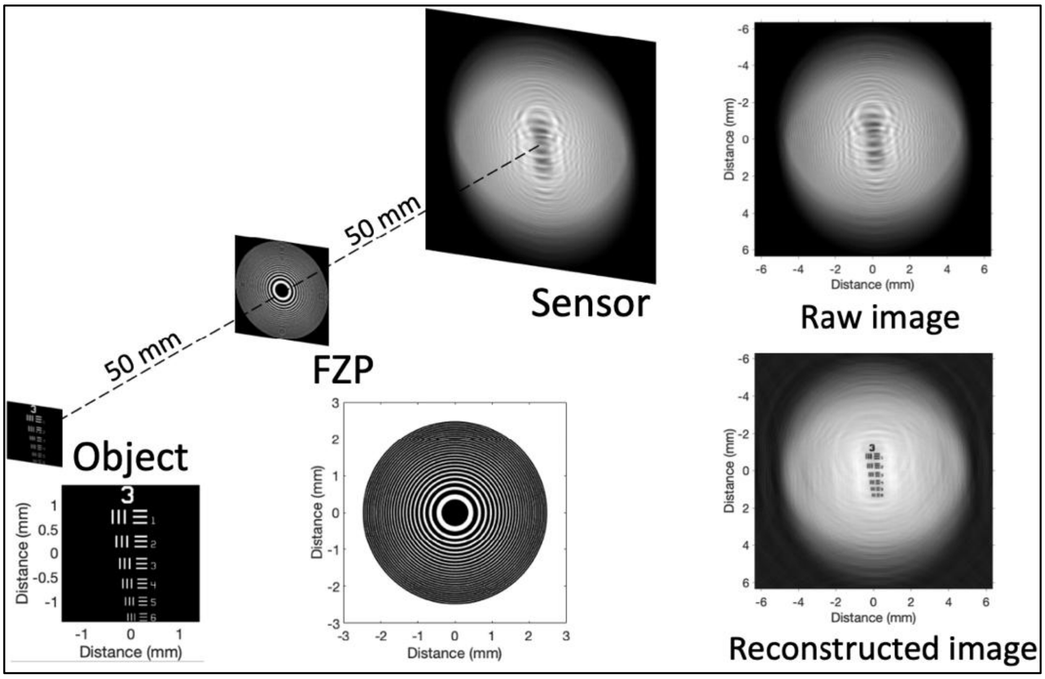

Figure S6 Ray-tracing method (direct approach) to theoretically generate a raw image of the 1951USAF target resolution's Group 3, considering the same object-FZP & FZP-sensor distances, field of view size, as well as FZP and sensor characteristics as those experimentally employed, except that the FZP was infinitely thin while its contrast material was entirely opaque.

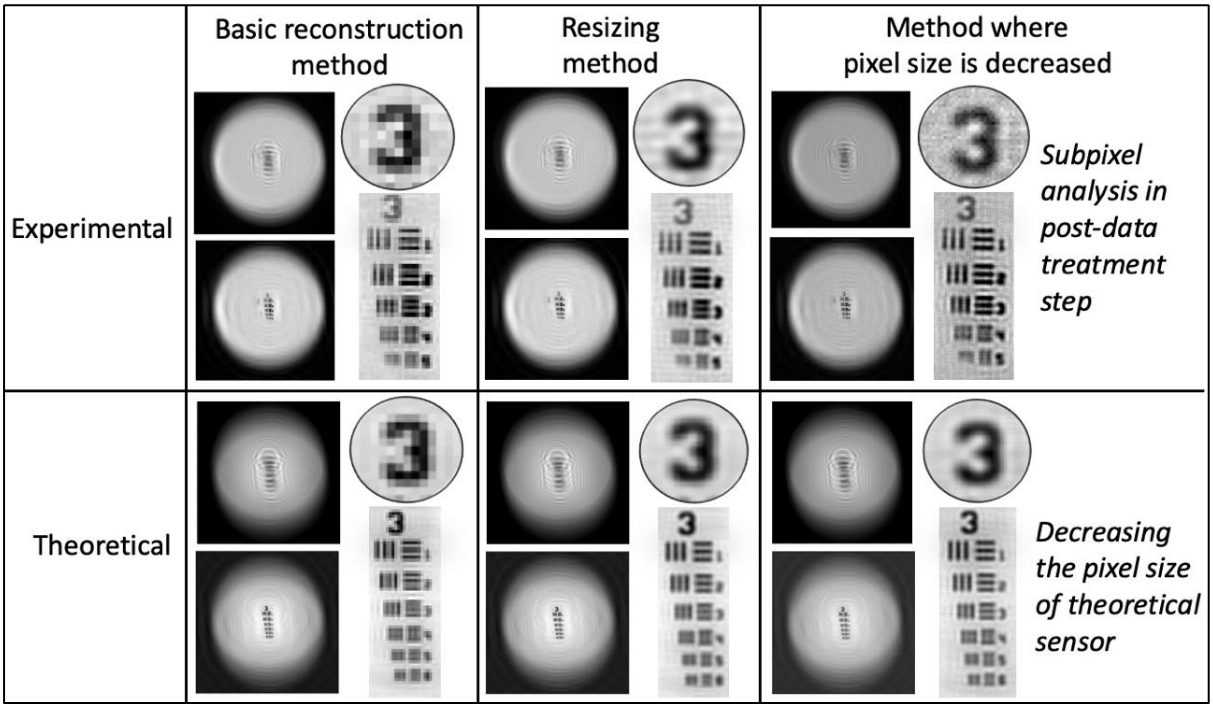

Figure S7 Comparison of results obtained using each of the three data-treatment approaches employed in this study: the basic reconstruction method (first column), the method consisting in enlarging the raw image prior to reconstruction (second column), and the method where the size of the sensor pixel is decreased (third column), using a raw image obtained experimentally (upper row) or theoretically using a ray-tracing approach described in the previous SI Figure (lower row).

S4. Results: chromium concentrations in the filter membranes

The measured Cr concentration retained on the dried membrane initially dipped into the 100% Cr(III) or Cr(VI) solution was 2.2 ± 0.2 or 2.0 ± 0.2 w. %, respectively. Given these concentrations, as well as the thickness (110 µm) and composition (polyamide) of the filter membranes, the expected absorption length after edge and edge-jump for these samples were less than 0.12 and 0.041, respectively. Therefore, performing hyperspectral XANES mapping would have been difficult on these thin membranes using a full-field imaging approach in transmission mode, since the theoretical absorption length after edge and edge-jump for optimum transmission should be around 2 and 1, respectively (Calvin, 2013). This was experimentally verified: when the filter membrane containing the highest Cr concentration (i.e. the one dipped into a 0.1 M Cr(III) solution) was analyzed at the bulk scale, noisy XANES spectra, which were not exactly reproducible from iteration to iteration, were acquired in transmission mode, whereas good quality and reproducible XANES spectra were acquired in fluorescence mode using a 13-pixel Silicone Drift Detector (SDD) fluorescence detector (Mirion) (Figure S8). No sign of self-absorption was observed when comparing the XANES spectra acquired in transmission and fluorescence mode on the same sample (Figure S8).

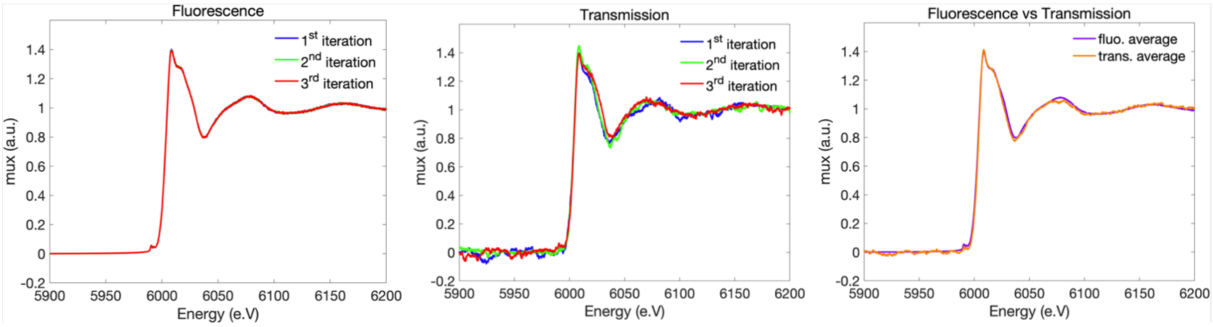

(A) (B) (C)

Figure S8 XANES spectra, corresponding to the filter membrane dipped into a 0.1 M Cr(III) solution, acquired at the bulk scale and in A) fluorescence mode (3 successive XANES iterations are shown); B) transmission mode (3 successive XANES iterations are shown); and C) the average of the three iterations acquired in each mode. Since these two average spectra overlapped very well, it could be concluded that no self-absorption phenomenon occurred when the spectra shown in A were collected in fluorescence mode, and thus also during our hyperspectral XANES mapping trial.

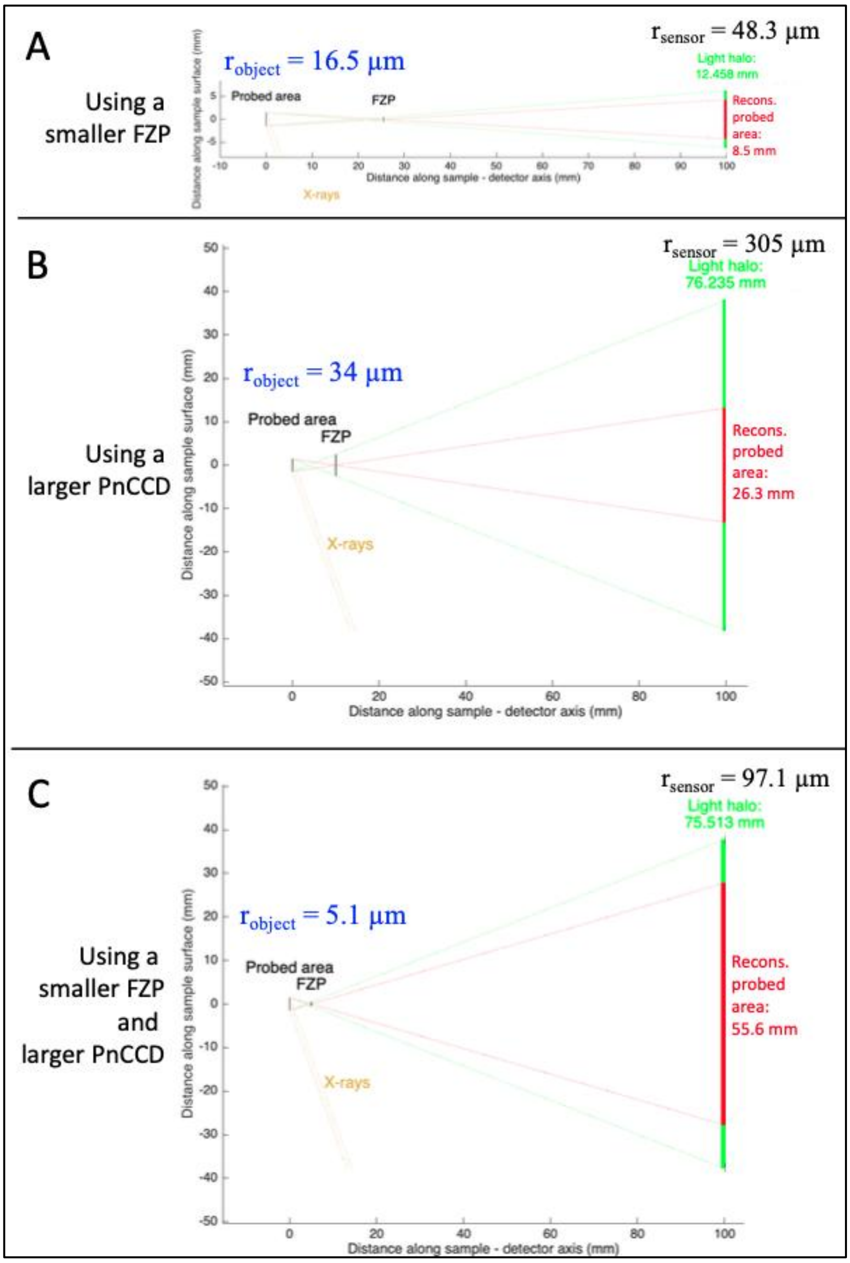

Figure S9 Spatial resolution achieved at object and sensor planes using experimental setups modified from the one employed in this study, using: (A) a smaller FZP, B) larger sensor, or C) both a smaller FZP & larger sensor.

S5. Methods: extraction of the XANES spectra

The "Reconstruction Input Frame", introduced in Methods and whose corresponding data was collected at a specific X-ray energy within the range of the XANES spectrum, was firstly resized by the resizing factor and the image reconstruction was then performed. Both the resizing & reconstruction steps were achieved using the algorithm supplied at the end of this document.

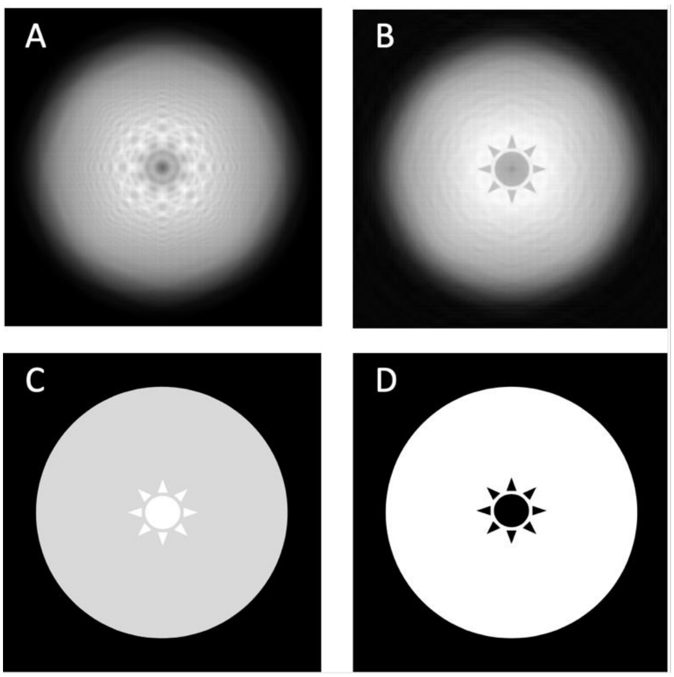

Figure S10 Theoretical image of an object, (A) before, and (B) after reconstruction. In the reconstructed image, the pixels corresponding to the object have, on average, intensities lower than those corresponding to the light halo outside of the object, as schematized in the sketch in (D). The pixel intensities do not follow a distribution as the one schematized in the sketch in (C), where the pixels corresponding to the object have the brightest intensity in the reconstructed image.

Due to the reconstruction method employed in this study, the pixel intensities, corresponding to the object featured in the reconstructed image, were in average lower than those located in the bright light halo outside of the object (Figure S10). Since the goal was to extract XANES spectra from multiple reconstructed images collected at specific X-ray energies, one could not simply invert the pixel intensities in each reconstructed image. Indeed, the differences in intensity of the pixels corresponding to the light halo (background pixels) between two reconstructed images collected at two successive X-ray energies within the XANES energy range could be much higher than the difference in intensity between the pixels corresponding to the object and those outside of it, in a given reconstructed image

collected at a specific X-ray energy. Therefore, the pixel intensities in each reconstructed image were modified using the pixel intensities from the raw image, as they were not affected by the reconstruction process. Specifically, each pixel intensity of the reconstructed image was divided by the sum of pixel intensities of the object in this image, and multipled by the sum of intensities of the raw image.

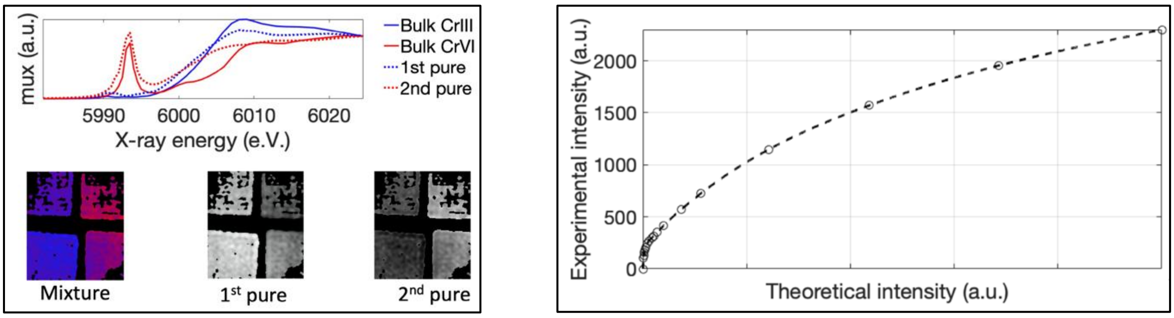

(A) (B)

Figure S11 A) Same Multivariate Curve Resolution - Alternating Least Squares (MCR-ALS) results as those shown in Figure 6 of the main manuscript, but for the case where no pixel intensity rescaling was done prior to performing the MCR-ALS procedure; and B) curve employed to rescale the experimental intensities of all pixels in each reconstructed image

Then, the pixel intensities were corrected for the non-linear response of the detector. If no pixel intensity rescaling was done prior to performing the MCR-ALS procedure, the absorption edges in the XANES spectra corresponding to the two pure species extracted by MCR-ALS occurred at lower energy than those in the XANES reference spectra of Cr(III) and Cr(VI) measured at the bulk scale (Figure S11A). This suggested that the quantification of photons was not linear during data collection, as such phenomenon can typically result in a compression and/or suppression of spectral features in the upper part of XANES spectra, which in turn inevitably results in a pull up of the lower part of the XANES spectra. This effect thus may give the impression that absorption edges are shifted to lower energies in the XANES spectra, as observed in Figure S11A. A correction was then applied to the intensities of all pixels in each reconstructed image, by rescaling them based on the curve shown in Figure S11B. This curve was empirically obtained by fitting a smoothing spline to spline knots whose coordinates were manually optimized. While the curve shown in Figure S11B indisputably allowed to eventually obtain MCR-ALS results, reported in Figure 6 of the main manuscript, that were much more realistic than those obtained when no intensity correction was performed (Figure S11A), this approach inevitably introduced a systematic error. It was thus believed that the pixel intensity rescaling approach represented one of the main reasons why the XANES spectra corresponding to the main species did not exactly match those expected.

Additionally, to identify in all reconstructed images the pixel locations where XANES spectra could be extracted, the mean in pixel intensities, recorded in all reconstruction images collected with an incident X-ray energy from 6010 to 6030 eV (i.e. above the Cr K edge at 5989 eV), was calculated at each pixel location. At each pixel location where this mean was below a specific threshold, all pixel intensities featured in the reconstruction images recorded at all X-ray energies were zeroed-out. The threshold was empirically determined based on the expected distribution of the pixels corresponding to the sample inside the field of view. For example, the threshold value employed in Figure S12A allowed us to successfully differentiate most sample pixels from the background pixels. However, some pixel locations corresponding to the background, far away from those corresponding to the sample in the reconstructed image, also had their pixel mean intensity recorded from 6010 to 6030 eV above this threshold (Figure S12A). Employing a higher threshold value to zero-out all pixel intensities corresponding to the background was not adequate since it also had the effect of zeroing-out most pixels corresponding to the sample (Figure S12B).

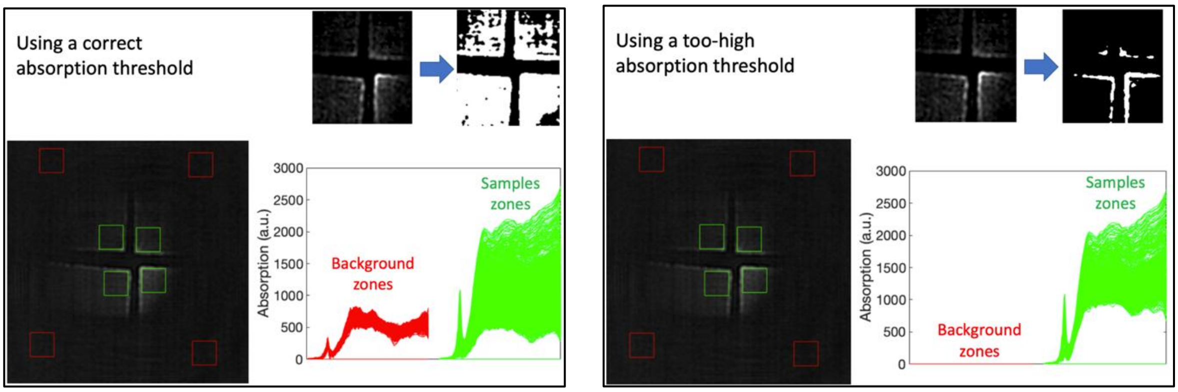

(A) (B)

Figure S12 Effect of thresholding using: (A) an adequate, or (B) too-high threshold when identifying the pixel locations where XANES can be extracted. In (A) & (B), four regions corresponding to sample and background areas, and the XANES spectra extracted at pixel locations inside these regions, are shown in green and red color, respectively.

The XANES spectra extracted from the background area, including four regions corresponding to the background shown in Figure S12A and located in the reconstructed image far from the region corresponding to the sample, seemed to correspond to a mixture of both Cr(III) and Cr(VI), based on their pre-edge feature heights (Figure S12A). This implied that the XANES of the background contributed to the XANES spectra extracted at pixel locations corresponding to the sample, including

those shown in green color in Figure S12A. This precluded from successfully mapping regions of the sample containing entirely Cr(III) or Cr(VI). Indeed, since the background provided a contribution of both Cr(III) & Cr(VI), a sample region that would contain only Cr(III) or Cr(VI) would also falsely feature Cr(VI) or Cr(III), respectively. Consequently, each individual XANES spectrum extracted in the pixel region corresponding to the sample within the reconstructed images was subtracted by the mean of the background XANES spectra, which were extracted from a pixel region outside of the pixel region corresponding to the sample. This approximation assumes that the same background spectrum, and at the same intensity level, contributes to all pixel intensities of the reconstructed images. This is not rigorously true since the height of the edge jumps featured in multiple XANES spectra extracted in four background regions could be different from each other (Figure S12A). Therefore, this represented an additional source of error in the results obtained from the XANES hyperspectral mapping approach. The fact that many pixels had intensities at pixel locations corresponding to the Cr-bearing membranes at the same levels as those corresponding to the background, outside of the membranes, was believed to be again a consequence of the non-linearity behavior of the detector, as such phenomenon may result in a decrease in pixel intensities. This is exactly what we observed in other unambiguous observations as previously mentioned. The reconstruction parameters, masking thresholds, and detector-response corrections were applied uniformly across all XANES energies and all regions of the sample image.

Finally, all extracted XANES spectra where smoothed using a Savitzky-Golay filter between 5997 and 6060 eV.

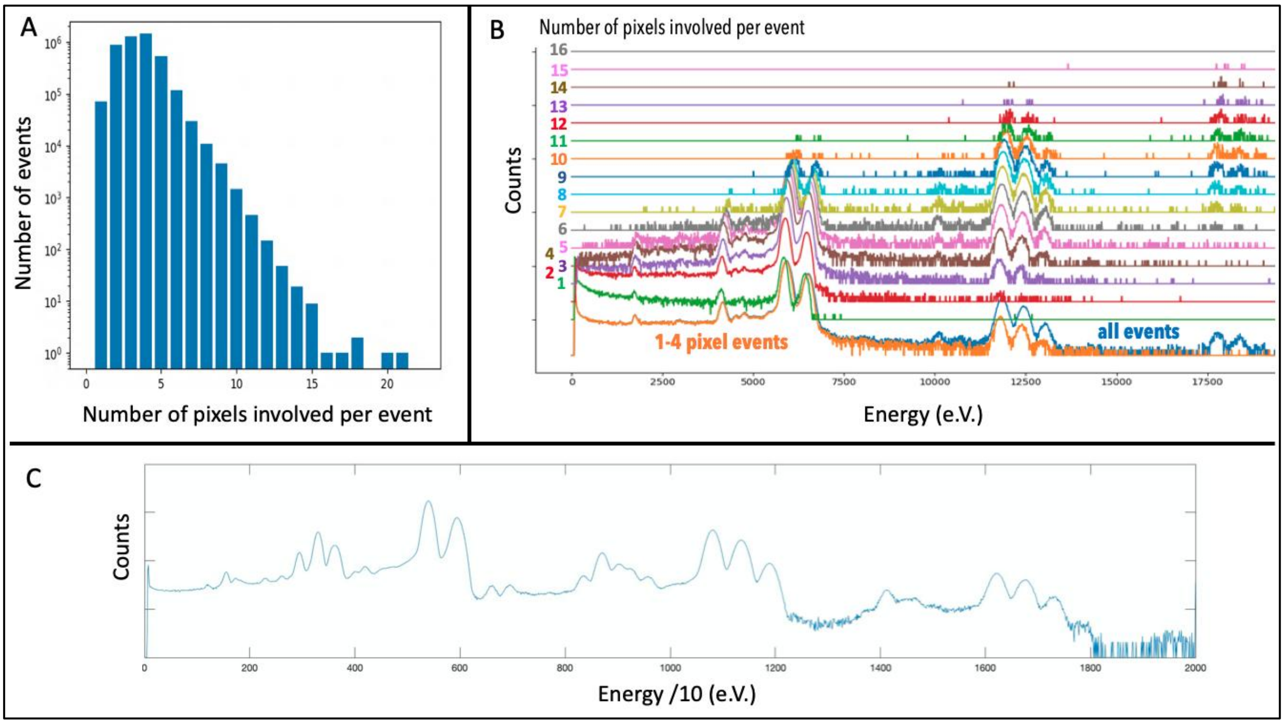

Figure S13 Laboratory measurement performed with the PnCCD at 32 kcps using a Fe55 source: (A) semi-log histogram of event size distribution, where the events are sorted based on the total number of pixels involved in the event (B) waterfall plot of individual spectra per event type (i.e. total number of pixels involved in each event), including at the bottom, the sum of all pixel event spectra or only 1- 4 pixel event spectra. While the sum of all events with size 5 or larger represented 15.9 % of all detected events during the present laboratory measurements, this ratio of rejected event, when only sizes 1-4 are used, should be higher at higher intensities. (C) Sum of all spectra collected at 69696 pixels (264 x 264 $pixels^2$) during the hyperspectral XANES mapping experiment, with the energy of the X-ray synchrotron beam at 6010 e.V. With the digital filter that excluded events corresponding to more than 4 pixels per event, the “apparent” count rate was 1203 kcps based on the raw merge spectrum not corrected for pile-ups. Two-fold and three-fold pile-ups could be indeed observed in the merge spectrum (C). These events corresponded to Cr photons that hit the sensor within the same 4 pixels during collection of a single frame, which was acquired using a 400 fps frame-rate. Overall, these results suggested that a significant amount of photons was filtered out during the hyperspectral XANES mapping trial, which corresponded to events involving at least from 5 to 15 pixels per event --it was, most likely, much more pixels per event than that, given the differences in count rates and magnitude of pile-ups between the laboratory experiment (A & B) & hyperspectral XANES mapping trial (C).

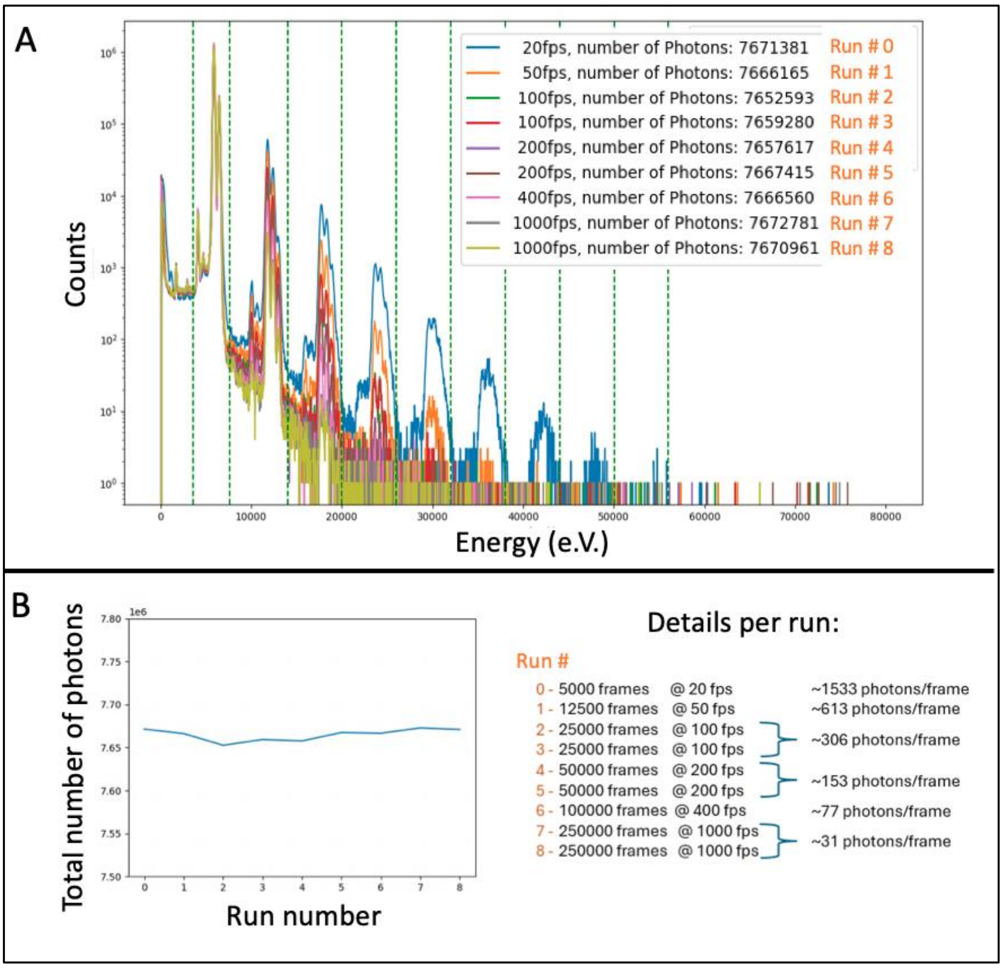

Figure S14 Laboratory measurement performed with the PnCCD using a Fe55 source, and without digital filtering of the events corresponding to more than 4 pixels per event : (A) spectra obtained with a constant total photon count (about 7.6 million photons) and decreasing frame rate, thus increasing counts per frame and therefore the pile-up probability, which effectively mimics higher count rates; and (B) total number of photons obtained from each spectrum shown in (A), after a pile-up correction approach was applied to the spectrum. This demonstrated that a linear response of the detector can be achieved at these count rates (i.e. the output photon number, processed by the detector, equates the input photon number reaching the detector) when a pile-up correction approach is applied to the unfiltered raw spectrum.

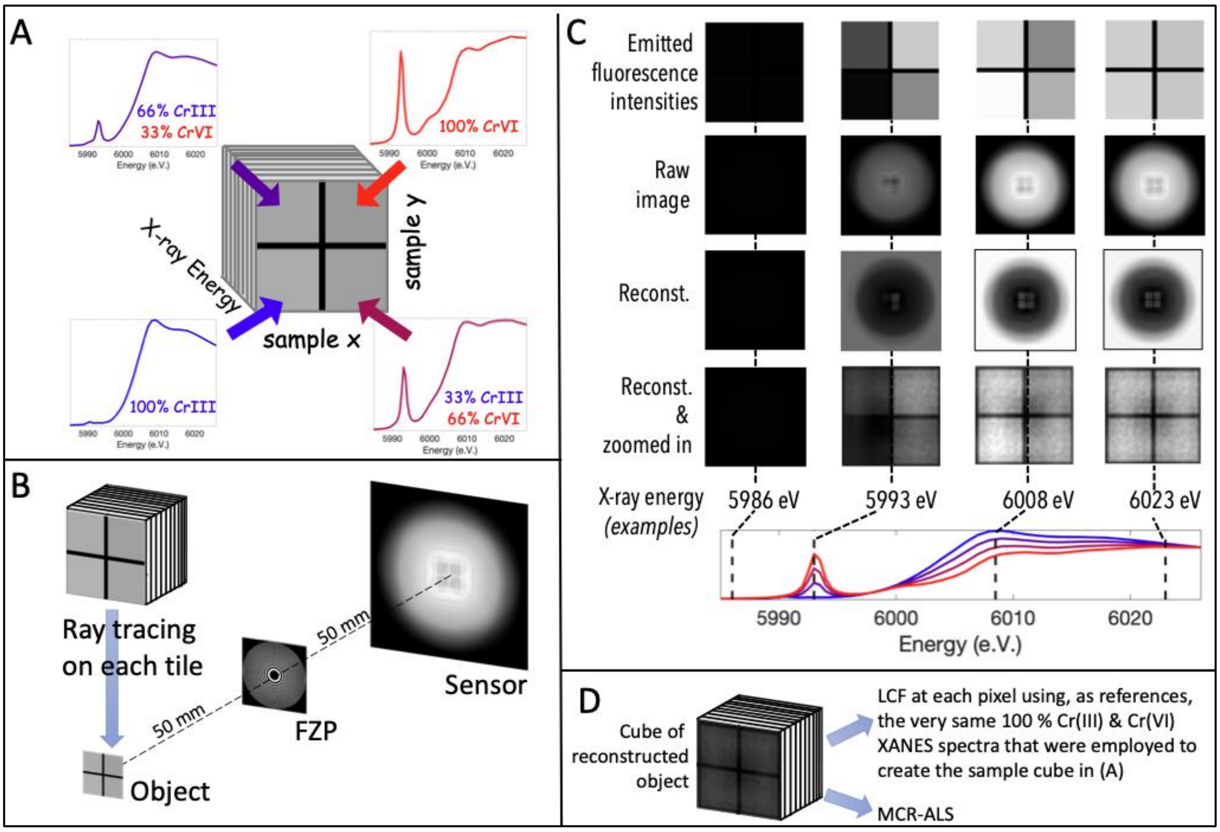

Figure S15 Theoretical hyperspectral XANES mapping: (A) theoretical 3D object, with four different sample regions in the X-Y plan corresponding to four specific Cr(III)-Cr(VI) mixture XANES spectra. These were created using the XANES spectra corresponding to the individual membrane chuncks exposed to the 100% Cr(III) or Cr(VI) solution, which were collected at the bulk scale and at the end of the actual hyperspectral experiment; (B) ray-tracing method (direct approach) to theoretically generate raw images for each tile of the sample cube shown in (A), considering the same object-FZP & FZP-sensor distances, field of view size, as well as FZP and sensor characteristics as those experimentally employed, except that the FZP was infinitely thin while its contrast material was entirely opaque; (C) examples, at selected energies within the XANES energy range, of raw & reconstructed images obtained with the ray-tracing approach; (D) data processing of the reconstructed object derived from ray-tracing.

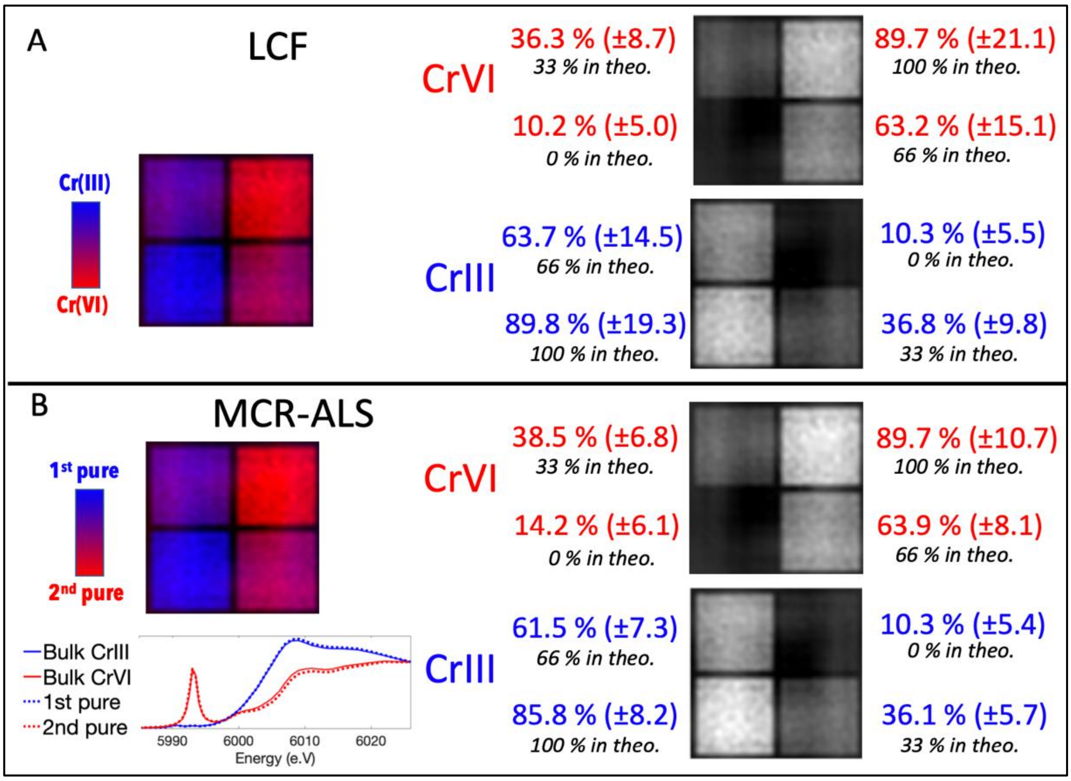

Figure S16 Results from the theoretical XANES mapping method described in Figure S15: (A) results obtained by LCF analyses, which were performed using, as references, the same two XANES spectra that were employed to create the theoretical sample cube, as detailed in Figure S14A ; and (B) results from MCR-ALS. The workflow corresponding to these data analyses is described in Figure S17.

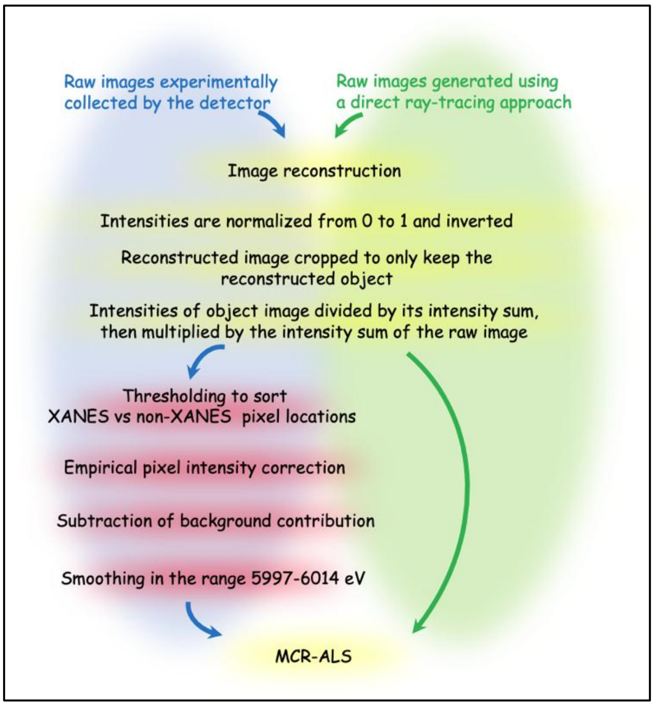

Figure S17 Workflow summarizing the steps to process the dataset acquired from the experimental hyperspectral XANES mapping experiment, or the theoretical hyperspectral XANES mapping recreation described in Figure S15. The steps shown in red color may not be mandatory if the detector response was linear, as it was the case with the dataset generated theoretically via a ray-tracing approach.

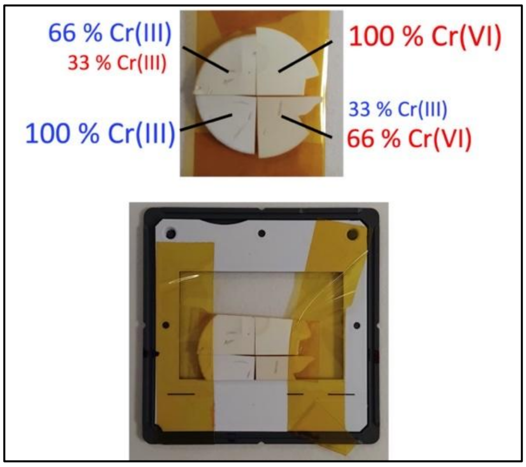

Figure S18 The sample (4 chunks of filter membranes impregnated with a specific Cr solution) used for the hyperspectral XANES mapping experiment before (top) and after (bottom) mounting it to a slide mount.

References

Calvin, S. (2013). *XAFS for Everyone*. Boca Raton: CRC Press.

Nowak, S. H., Bjeoumikhov, A., von Borany, J., Buchriegler, J., Munnik, F., Petric, M., Radtke, M., Renno, A. D., Reinholz, U., Scharf, O. & Wedell, R. (2015). *Journal of Analytical Atomic Spectrometry* **30**, 1890-1897.

Wu, J., Zhang, H., Zhang, W., Jin, G., Cao, L. & Barbastathis, G. (2020). *Light: Science & Applications* **9**, 53.

PYTHON RECONSTRUCTION SCRIPT:

```
import numpy as np
from PIL import Image
from scipy.fft import fftfreq, fft2, ifft2
from scipy.special import erfc
from imresize import imresize

def FZP_CodedAperture_reconstruction(raw_image,FZP_r1,FZP_radius,sensor_pixel_size,
dist_sample_FZP,dist_sample_sensor,field_of_view_length):

    ## This code is a modified version, and adapted to Python, of the Matlab scripts "phaserec_ctf.m" &
    ## "ctfRegWeights.m" belonging to the "HoloTomoToolbox", from the Lohse et al. manuscript "A
phase-retrieval toolbox for X-ray
    ## holography and tomography", J. Synchrotron Rad. (2020). 27, 852–859.
    ##
    ## The main modifications are described in the Landrot et al. manuscript "Full-field X-ray
    ## fluorescence imaging using a Fresnel Zone Plate as a coded aperture:
    ## optimized reconstruction algorithm and first trial of hyperspectral XANES mapping" (2026). The
script below is provided as supplementary
    ## information of the Landrot et al. study.
    ##
    ##
    ## ## Prerequisite:
```

One important modification of the original reconstruction approach is the added image resizing step prior performing the reconstruction as

discussed in the Landrot et al. 2026 study. This is achieved in the Matlab version of this script using the function "imresize.m" from the Matlab Image

Processing Toolbox. Its equivalent Python function "imresize.py", from Aleksandr Petiushko (available here: https://github.com/fatheral/matlab_imresize)

is employed in the code below.

##

Function input parameters:

"raw_image" is a 2x2 array corresponding to the raw image collected by the sensor

"FZP_r1" is the radius of the FZP inner zone, in mm

"FZP_radius" is the radius of the entire FZP, in mm

"sensor_pixel_size", is the length of the edge of a square pixel of the sensor, in mm

"dist_sample_FZP", is the distance between the sample and the FZP, in mm

"dist_sample_sensor", is the distance between the sample and the bidimentional detector's sensor, in mm

"field_of_view_length" is the length of the square edge of the field of view, which is assumed in this code to have a square shape

##

Example:

If a raw image, saved as a .tiff file, was taken from an object illuminated within a 3 x 3 mm2

field of view (e.g. case of a 1 x 3 mm2 (H xV) X-ray beam hitting the sample

plane tilted by a 20-degree angle relative to beam path), using a FZP and sensor at 50 and

100mm from the sample, respectively, with a 48-micrometer sensor pixel

size, a FZP featuring a 5mm diameter and 0.354 mm inner radius, then, a Python script calling this function could be:

import numpy as np

import matplotlib.pyplot as plt

import tifffile as tiff

from PIL import Image

from FZP_CodedAperture_reconstruction import FZP_CodedAperture_reconstruction

Step1: Importing data

I = np.array(Image.open('my_raw_image.tiff').convert('L'))

Step2: Calling this reconstruction function to reconstruct the object image

result = FZP_CodedAperture_reconstruction(I,0.354,2.5,0.048,13.9,50,3)

Step3: Rescaling intensities so that the image can be displayed

im_size = np.array(result.shape)

```
## A = np.zeros((im_size[0], im_size[1], 3))
## A[:, :, 0] = result2
## A[:, :, 1] = result2
## A[:, :, 2] = result2
## fig = plt.figure()
## plt.imshow(A)
## plt.show()
##
## ##  Note 1:
## The regularization parameter is determined with the lower and upper limits equal to 1e-2 & 1e-1,
respectively.
## These two default values can be modified at lines 116 and 117 below.
##
## ##  Note 2:
## This reconstruction function can be easily inverted to a script
## that would allow obtaining the corresponding raw image from a given
## reconstructed image. This would be achieved by:
## Step 1: replacing the "raw_image" input parameter by "reconstructed_image", which would be a
2x2 array
## corresponding to the reconstructed image.
## Step 2: deleting lines 88 -90 included
## Step 3: at line 97, 114 & 124 below, replacing "resized_image" by
## "reconstructed_image", i.e. NN = np.array(reconstructed_image.shape),
## sizeHolo=np.array(reconstructed_image.shape), & XiSqDivFresnel =
np.zeros(reconstructed_image.shape).astype(np.float64); respectively.
## Step 4: replacing the main equation (line 132 below) by:
## raw_image=np.real(ifft2((fft2(reconstructed_image)*(CTF**2+ regWeights ))/CTF));
## Step 5: replacing the last four lines by:
## # Rearranging raw image
## raw_image = np.flip(raw_image,axis=1)
## return raw_image


# Determining at sensor position the width of light halo forming the raw image
halo_width=2*np.sqrt((((FZP_radius-(-
field_of_view_length/2))/dist_sample_FZP)*(dist_sample_sensor)+(-field_of_view_length/2))**2);
```

```
    # Determining the factor employed to enlarge the raw image, as discussed in the Landrot et al. 2025 manuscript
    Resizing_Factor=halo_width/field_of_view_length;

    # Enlarging the raw image, as discussed in the Landrot et al. 2025 manuscript
    image_shape = np.array(raw_image.shape)
    resized_image= imresize(raw_image,output_shape=(image_shape[0]*Resizing_Factor, image_shape[1]*Resizing_Factor))

    # Determining the Fresnel number (N)
    M=dist_sample_sensor/dist_sample_FZP
N=((sensor_pixel_size/Resizing_Factor)*(sensor_pixel_size/Resizing_Factor))/((FZP_r1*M)*(FZP_r1*M))

    # Determining coordinates in Fourier space
    NN = np.array(resized_image.shape)
    dx = 1
    dx = np.expand_dims(dx, axis=0) * np.ones([1,len(NN)])
    ndim = len(NN)
    xi=[np.fft.ifftshift(np.arange(-np.floor(0.5*NN[jj]), np.floor(0.5*(NN[jj]-1))+1).T * (2*np.pi / (NN[jj]*dx[0,jj])))
    for jj in range(ndim)]
    xi_y = xi[0]
    xi_x = xi[1].T

    # Determining the Contrast Transfer Function (CTF)
    v1=xi_y** 2 / (4 * np.pi * N)
    v2=xi_x** 2 / (4 * np.pi * N)
    v3=np.transpose(np.array([v1]*v2.size));
    chi=v2+v3
    CTF = np.cos(chi)

    # Determining the regularization parameter (regWeights)
    sizeHolo=np.array(resized_image.shape)
    fresnelNumbers=np.mean(N * np.ones(2));
    lower_limit=1e-2;
```

```
    upper_limit=1e-1;
    xi = [None, None]
    dx = 1
    dx = dx * np.ones(2)
    for jj in range(2):
        xi[jj] = np.fft.ifftshift((np.arange(-np.floor(0.5 * sizeHolo[jj]), np.floor(0.5 * (sizeHolo[jj] - 1)) +
1) * (2 * np.pi / (sizeHolo[jj] * dx[jj]))))
    xi[1]=xi[1].reshape(-1,1)
    XiSqDivFresnel = np.zeros(resized_image.shape).astype(np.float64);
    for jj in range(2):
        XiSqDivFresnel += xi[jj] ** 2 / (2 * np.pi ** 2 * fresnelNumbers)
    sigma = (np.sqrt(2) - 1) / 2
    w = 1 / 2 * erfc((np.sqrt(XiSqDivFresnel) - 1) / (np.sqrt(2) * sigma))
    regWeights = lower_limit * w + upper_limit * (1 - w)

    # Performing the image reconstruction
    reconstructed_image = np.real(ifft2((CTF * fft2(resized_image)) / (CTF ** 2 + regWeights)))

    # Rearranging reconstructed image
    reconstructed_image = np.flip(reconstructed_image,axis=1)

    return reconstructed_image
```

MATLAB RECONSTRUCTION SCRIPT:

```
function reconstructed_image=FZP_CodedAperture_reconstruction(raw_image,
FZP_r1,FZP_radius,sensor_pixel_size,dist_sample_FZP,dist_sample_sensor,field_of_view_length,var
argin)

% This code is a modified version of the Matlab scripts "phaserec_ctf.m" &
% "ctfRegWeights.m" belonging to the "HoloTomoToolbox", from the Lohse et al. manuscript "A
phase-retrieval toolbox for X-ray
% holography and tomography", J. Synchrotron Rad. (2020). 27, 852–859.
%
% The main modifications are described in the Landrot et al. manuscript "Full-field X-ray
% fluorescence imaging using a Fresnel Zone Plate as a coded aperture:
% optimized reconstruction algorithm and first trial of hyperspectral XANES mapping" (2026). The
script below is provided as supplementary
% information of the Landrot et al. 2026 study.
%
%
% %%% Prerequisite:
% A valid Matlab License is required to run this code. It also requires the
% Matlab Image Processing toolbox to call the function "imresize".
% The script was successfully tested on a MATLAB Version: 9.14.0.2254940
% (R2023a) Update 2.
%
% %%% Function input parameters:
% "raw_image" is a 2x2 array corresponding to the raw image collected by the sensor
% "FZP_r1" is the radius of the FZP inner zone, in mm
% "FZP_radius" is the radius of the entire FZP, in mm
% "sensor_pixel_size", is the length of the edge of a square pixel of the sensor, in mm
% "dist_sample_FZP", is the distance between the sample and the FZP, in mm
% "dist_sample_sensor", is the distance between the sample and the bidimentional detector's sensor, in
mm
% "field_of_view_length" is the length of the square edge of the field of view, which is assumed in
this code to have a square shape
% The built-in Matlab varargin input parameter allows to add, optionally,
% specific values for the lower and upper limits of the regularization parameter.
% If these two optional values are not provided, the default values of
```

```
% the lower & upper limits are 1e-2 & 1e-1, respectively.
%
% %%% Example:
% If a raw image, saved as a .tif file, was taken from an object illuminated within a 3 x 3 mm2
% field of view (e.g. case of a 1 x 3 mm2 (H xV) X-ray beam hitting the sample
% plane tilted by a 20-degree angle relative to beam path), using a FZP and sensor at 50 and
% 100mm from the sample, respectively, with a 48-micrometer sensor pixel
% size, a FZP featuring a 5mm diameter and 0.354 mm inner radius, then, a Matlab script calling this
function could be:
% % Step1: Importing data
% raw_image=double(importdata('my_raw_image.tif'));
% % Step2: Calling this reconstruction function to reconstruct the object image
% reconstructed_image=FZP_CodedAperture_reconstruction(raw_image,0.354,2.5,0.048,50,100,3);
% % Step3: Rescaling intensities so that the image can be displayed
% reconstructed_image=reconstructed_image-min(min(reconstructed_image));
% reconstructed_image=reconstructed_image/max(max(reconstructed_image));
% % Step4: Displaying reconstructed image
% figure,imshow(reconstructed_image)
%
% %%% Note 1:
% In the same example above, if specific values other than the default
% ones are provided for the lower and upper limits of the regularization
% parameter, e.g. 5e-3 & 2e-1, then the calling of the reconstruction function should be:
% reconstructed_image=FZP_CodedAperture_reconstruction(raw_image,0.354,2.5,0.048,50,100,3,5e-
3,2e-1);
%
% %%% Note 2:
% This reconstruction function can be easily inverted to a script
% that would allow obtaining the corresponding raw image from a given
% reconstructed image. This would be achieved by:
% Step 1: replacing the "reconstructed_image" output parameter by
% "raw_image", and similarly, replacing the "raw_image" input parameter by "reconstructed_image",
which would be a 2x2 array
% corresponding to the reconstructed image.
% Step 2: deleting lines 79 -81 included
% Step 3: at line 88 & 101 below, replacing "resized_image" by
% "reconstructed_image", i.e. NN=size(reconstructed_image) &
```

```
% sizeHolo=size(reconstructed_image), respectively.
% Step 4: replacing the main equation (line 126 below) by:
% raw_image=ifft2((fft2(reconstructed_image).*(CTF.^2+ regWeights ))./CTF);
% Step 5: replacing the last line by: raw_image=flip(raw_image,2);


format long g

% Determining at sensor position the width of light halo forming the raw
% image
halo_width=2*sqrt((((FZP_radius-(-
field_of_view_length/2))/dist_sample_FZP)*(dist_sample_sensor)+(-field_of_view_length/2))^2);

% Determining the factor employed to enlarge the raw image, as discussed in the Landrot et al. 2025
manuscript
Resizing_Factor=halo_width/field_of_view_length;

% Enlarging the raw image, as discussed in the Landrot et al. 2025 manuscript
[row,col]=size(raw_image);
resized_image = imresize(raw_image,[row*Resizing_Factor col*Resizing_Factor]);

% Determining the Fresnel number (N)
M=dist_sample_sensor/dist_sample_FZP;
N=((sensor_pixel_size/Resizing_Factor)*(sensor_pixel_size/Resizing_Factor))/((FZP_r1*M)*(FZP_r
1*M));

% Determining coordinates in Fourier space
NN=size(resized_image);
ndim = numel(NN);
xi = cell(1, ndim);
for jj = 1:ndim
   xi{jj} = ifftshift( ( -floor(0.5*NN(jj)) : floor(0.5*(NN(jj)-1)) ).' * ( 2*pi / (NN(jj)) ),1 );
end
xi_y=xi{1};
xi_x=xi{2}';

% Determining the Contrast Transfer Function (CTF)
```

```
CTF =   cos(xi_y.^2/(4*pi*N) + xi_x.^2/(4*pi*N));

% Determining the regularization parameter (regWeights)
sizeHolo=size(resized_image);
fresnelNumbers=mean(N*ones(2,1),2);
if isempty(varargin)
    lower_limit=1e-2;
    upper_limit=1e-1;
else
    lower_limit=varargin{1};
    upper_limit=varargin{2};
end
xi = cell([2,1]);
dx = 1;
dx = dx(:).' .* ones([1,2]);
for jj = 1:2
 xi{jj} = ifftshift( ( -floor(0.5*sizeHolo(jj)) : floor(0.5*(sizeHolo(jj)-1)) ).' * ( 2*pi / (sizeHolo(jj)*dx(jj)) ) );
end
 xi{2} = xi{2}';
XiSqDivFresnel = 0;
for jj = 1:2
    XiSqDivFresnel = XiSqDivFresnel + xi{jj}.^2 ./ (2*pi^2*fresnelNumbers(jj));
end
sigma = (sqrt(2) - 1) / 2;
w = 1/2 * erfc((sqrt(XiSqDivFresnel) - 1) / (sqrt(2) * sigma));
regWeights = lower_limit * w + upper_limit * (1 - w);

% Performing the image reconstruction
reconstructed_image= ifft2((CTF.*fft2(resized_image)) ./ (CTF.^2+ regWeights ));

% Rearranging reconstructed image
reconstructed_image=flip(reconstructed_image,2);
```
